# Effectiveness of eHealth Interventions on Moderate-to-Vigorous Intensity Physical Activity Among Patients in Cardiac Rehabilitation: Systematic Review and Meta-analysis

**DOI:** 10.2196/42845

**Published:** 2023-03-29

**Authors:** Tianzhuo Yu, Haiyan Xu, Xin Sui, Xin Zhang, Yue Pang, Tianyue Yu, Xiaoqian Lian, Ting Zeng, Yuejin Wu, Xin Leng, Feng Li

**Affiliations:** 1 School of Nursing Jilin University Changchun China

**Keywords:** cardiac rehabilitation, cardiorespiratory fitness, cardiovascular diseases, exercise, physical activity, heart disease risk factors, meta-analysis, systematic review, eHealth, telemedicine

## Abstract

**Background:**

Cardiac rehabilitation is a class IA recommendation for patients with cardiovascular diseases. Physical activity is the core component and core competency of a cardiac rehabilitation program. However, many patients with cardiovascular diseases are failing to meet cardiac rehabilitation guidelines that recommend moderate-to-vigorous intensity physical activity.

**Objective:**

The major objective of this study was to review the evidence of the effectiveness of eHealth interventions in increasing moderate-to-vigorous intensity physical activity among patients in cardiac rehabilitation. The secondary objective was to examine the effectiveness of eHealth interventions in improving cardiovascular-related outcomes, that is, cardiorespiratory fitness, waist circumference, and systolic blood pressure.

**Methods:**

A comprehensive search strategy was developed, and a systematic search of 4 electronic databases (PubMed, Web of Science, Embase, and Cochrane Library) was conducted for papers published from the start of the creation of the database until November 27, 2022. Experimental studies reporting on eHealth interventions designed to increase moderate-to-vigorous intensity physical activity among patients in cardiac rehabilitation were included. Multiple unblinded reviewers determined the study eligibility and extracted data. Risk of bias was evaluated using the Cochrane Collaboration Tool for randomized controlled trials and the Cochrane Effective Practice and Organization of Care group methods for nonrandomized controlled trials. A random-effect model was used to provide the summary measures of effect (ie, standardized mean difference and 95% CI). All statistical analyses were performed using Stata 17.

**Results:**

We screened 3636 studies, but only 29 studies were included in the final review, of which 18 were included in the meta-analysis. The meta-analysis demonstrated that eHealth interventions improved moderate-to-vigorous intensity physical activity (standardized mean difference=0.18, 95% CI 0.07-0.28; *P*=.001) and vigorous-intensity physical activity (standardized mean difference=0.2, 95% CI 0.00-0.39; *P*=.048) but did not improve moderate-intensity physical activity (standardized mean difference=0.19, 95% CI –0.12 to 0.51; *P*=.23). No changes were observed in the cardiovascular-related outcomes. Post hoc subgroup analyses identified that wearable-based, web-based, and communication-based eHealth intervention delivery methods were effective.

**Conclusions:**

eHealth interventions are effective at increasing minutes per week of moderate-to-vigorous intensity physical activity among patients in cardiac rehabilitation. There was no difference in the effectiveness of the major eHealth intervention delivery methods, thereby providing evidence that in the future, health care professionals and researchers can personalize convenient and affordable interventions tailored to patient characteristics and needs to eliminate the inconvenience of visiting center-based cardiac rehabilitation programs during the COVID-19 pandemic and to provide better support for home-based maintenance of cardiac rehabilitation.

**Trial Registration:**

PROSPERO International Prospective Register of Systematic Reviews CRD42021278029; https://www.crd.york.ac.uk/prospero/display_record.php?RecordID=278029

## Introduction

### Background

Cardiovascular diseases (CVDs) are defined as diseases concerning the heart or blood vessels and are a range of disorders involving the circulatory system. CVDs are the leading cause of death globally [1] and are responsible for approximately 20% of the worldwide disease burden [2]. Cardiac rehabilitation (CR) is a class IA recommendation for people with CVD and can reduce mortality, morbidity, and unplanned hospital admissions as well as improve exercise capacity, quality of life, and psychological well-being [3]. The American Heart Association professional medical guidelines strongly recommend referral to CR after acute coronary syndrome, percutaneous coronary syndrome, coronary artery bypass surgery, and stable angina [4]. As a core component and core competency of CR programs [5], physical activity is one of the best lifestyle risk factor management strategies for patients to reduce the progression of CVD or death from CVD [6]. Physical activity is “any bodily movement produced by the skeletal muscle that requires energy expenditure” [7]. The World Health Organization recommends that adults and older adults (aged 18 years and older) with chronic conditions but without contraindications accumulate at least 150 minutes of weekly moderate-to-vigorous intensity physical activity (MVPA) to receive substantial health benefits [8]. Current CR guidelines recommend that patients with stable CVD engage in 30-60 minutes of moderate-intensity physical activity (MPA) (eg, brisk walking) at least 5 days per week (preferably 7 days per week), supplemented by increased daily lifestyle physical activity (eg, household work or walking during breaks from work) to improve cardiorespiratory fitness (CRF) [6,9]. However, previous observational studies demonstrated that many people with CVD fail to meet the recommended daily physical activity levels [10], and many patients in CR continue to be physically inactive despite participation in CR [11], with as little as 11 minutes per day of MVPA [12]. There is an urgent need for an effective method to supervise physical activity in patients with CVD who require CR. A systematic review of the impact of increased physical activity in the secondary prevention of coronary heart disease by Vasankari et al [13] found that new tools using web-based apps and smart devices are promising means of providing remote guidance for patients who are unable to participate in regular exercise sessions. Web-based apps and smart devices fall squarely into the category of eHealth. eHealth is defined broadly as “an emerging field in the intersection of medical informatics, public health and business, referring to health services and information delivered or enhanced through the internet and related technologies” [14] and provides a unique opportunity for the implementation of population-wide behavioral interventions targeting physical activity [15]. eHealth intervention technologies include telemedicine or telehealth, web-based strategies, email, mobile phones, mobile or smartphone apps, text messaging, digital games, and wearables or monitoring devices [16,17]. They are highly recommended for encouraging and tracking MVPA due to their low cost, high efficiency, and accessible data collection [18,19]. eHealth interventions showed promising results in CR, supporting physical activity improvement. For example, Ashur et al [20] reviewed the use of wearable activity trackers to promote physical activity among patients in CR and found that all 3 studies that included a pedometer or an accelerometer showed statistically significant improvements in daily step counts. Duan et al [21] conducted a meta-analysis of the impact of eHealth-based multiple health behavior change interventions on physical activity in people with noncommunicable diseases and showed that those interventions significantly promoted physical activity (standardized mean difference [SMD]=0.85, 95% CI 0.23-1.47; *P*=.008).

Physical activity is a key outcome measure of progress made by people participating in CR [22]. Few reviews have measured physical activity levels in patients in CR by counting the number of steps per day or leisure physical activity time instead of MVPA [23]. Recently, Patterson et al [23] completed a systematic review and meta-analysis on MVPA in people with CVD, but studies only included smartphone app interventions, which is only one branch of eHealth interventions. No study has specifically reported on the effects of more comprehensive eHealth interventions on MVPA among patients in CR. Therefore, to compare the effectiveness of eHealth interventions with that of non-eHealth interventions in improving MVPA in patients in CR, we performed this systematic review and meta-analysis.

CR improves survival in people with CVD, mediated mainly by improvements in CRF. CRF is an indicator of heart and body muscle function, and in addition to genetic factors, it largely reflects the level of physical activity [24]. There is a wide variety of methods to assess CRF, including peak oxygen uptake measured directly during cardiorespiratory exercise testing or the 6-minute walk test. Vanhees et al [25] demonstrated that peak oxygen uptake after exercise training remained a significant independent predictor for all-cause and cardiovascular mortality. Similarly, as also found by Carbone et al [26], post-CR peak oxygen uptake was a strong and independent predictor of long-term survival in people with coronary heart disease. Obesity is a risk factor for CVD, and it causes various metabolic diseases, particularly damage of the circulatory system [27]. Bastien et al [28] showed that waist circumference (WC) could represent central obesity and is essential for determining overall health and cardiovascular risks. Hypertension is among the most modifiable CVD risk factors, and successful control of systolic blood pressure (SBP) is recognized as essential to long-term cardiovascular health [29]. There is evidence that CR and exercise training produce marked benefits in CRF and cardiovascular risk factors such as obesity index and blood pressure [30]. Therefore, we selected CRF, WC, and SBP as cardiovascular-related secondary outcomes.

### Aims of This Review

The primary aim of this study was to systematically review and report on the evidence examining the effectiveness of eHealth interventions designed to increase MVPA among patients in CR. The secondary aim was to investigate the effectiveness of eHealth interventions in improving cardiovascular-related outcomes.

## Methods

### Overview

The methods were registered prospectively with PROSPERO (International Prospective Register of Systematic Reviews, registration CRD42021278029, registration date: September 17, 2021). This paper has been amended from the information provided at registration (Multimedia Appendix 1). This paper was prepared in adherence to the recommendations of the Cochrane Collaboration [31] and the PRISMA (Preferred Reporting Items for Systematic Reviews and Meta-Analyses) 2020 statement [32] (Multimedia Appendix 2).

### Eligibility Criteria

This review included studies reporting on eHealth interventions designed to increase MVPA among patients in CR.

#### Participants

Studies were included if participants were adults (≥18 years) with CVD who were eligible for CR. There were no restrictions on the other demographic data of the participants.

#### Interventions

eHealth was considered as any intervention that included at least one of the following components: wearable health and movement trackers, websites, smartphone apps, messaging services (ie, text messaging, emails), video games, or telehealth [18]. Studies that used single-component or multicomponent eHealth interventions as the stand-alone or the primary component for the intervention were included. Increasing MVPA was an aim of the intervention in each of the studies.

#### Comparators

Control groups were used, when available, to compare the effects between groups by using meta-analysis. The control groups included participation in the non–eHealth interventions (eg, usual care that follows standard CR exercise guidelines or center-based CR exercises).

#### Outcomes

According to the classification of physical activity intensity in the 2021 European Society of Cardiology Guidelines on CVD prevention in clinical practice, moderate intensity is defined as 3-5.9 metabolic equivalents of a task (METs) (eg, walking at a moderate or brisk pace, slow cycling, double tennis) and vigorous intensity is defined as ≥6 METs (eg, race walking, running, single tennis) [33]. The primary outcome was defined as the total amount of physical activity executed with at least moderate intensity per week measured either objectively (eg, accelerometers, pedometers) or by self-report (eg, questionnaires, diaries). “At least moderate-intensity” physical activity refers to physical activity of moderate intensity, vigorous intensity, and moderate-to-vigorous intensity. Because many studies do not measure MPA and VPA separately but rather measure the sum of MPA and VPA as a whole, that is, MVPA, our primary outcome indicators included not only MPA and VPA but also MVPA. Secondary outcomes included CRF, WC, and SBP.

#### Study Designs

Experimental studies (ie, multicenter randomized controlled trial [RCT], single-center RCT, cluster RCT, quasi-experimental, and pre-post studies) were eligible.

#### Publication Status and Language

Full-text research papers were eligible for this review, but conference abstracts, dissertations, and grey literature were ineligible. There were no restrictions on the language of publication.

### Information Sources

Four electronic databases (PubMed, Web of Science, Embase, and Cochrane Library) were searched from inception to November 27, 2022. In addition, reference lists of the included studies and relevant systematic reviews and meta-analyses identified by the search strategy were manually searched to identify additional studies.

### Search Strategy

The search strategy was designed around 4 subject areas: CR, eHealth, physical activity, and experimental studies (Multimedia Appendix 3). The key search terms included CR, eHealth, cell phone, text messaging, internet, mobile apps, web-based, computer-assisted instruction, computer-tailored, Wii, exercise video games, accelerometry, electronic mail, wearable devices, mHealth, telemedicine, physical activity, motor activity, exercise, physical fitness, physical education and training, exercise therapy, movement, bicycling, walking, running, yoga, moderate intensity activity, vigorous intensity activity, MVPA, program evaluation, evaluation study, multicenter study, observational study, RCT, clinical trial, controlled clinical trial, case-control studies, and cohort studies. This strategy was developed in PubMed and was modified for other databases.

### Selection Process

The search results were exported into the document management software EndNote 20.1, and duplicates were removed. Two independent reviewers, Tianzhuo Y and HX, screened the titles and the abstracts of each study to identify potentially relevant papers. In addition, the full texts of all the papers that met the inclusion criteria were obtained and independently reviewed. When disagreements between reviewers arose, discrepancies were resolved by FL, a third reviewer.

### Data Collection Process and Data Items

Two independent reviewers, Tianzhuo Y and XS, extracted and verified the data, and any cases of disagreement were arbitrated by FL. Data sheets were created in preestablished Microsoft Excel, which included publication details (first author, publication year, country), participants’ characteristics (age, gender, referral diagnosis), sample size, study design (multicenter RCT, single-center RCT, cluster RCT, quasi-experimental, pre-post), intervention, control description (eHealth component, content description of intervention group or control group), blinding and randomization techniques, methods of MVPA measurement (measurement tool, objective or self-reported measurement, units of measurement), MVPA data (baseline and postintervention mean differences, standard deviations, medians, interquartile range, and percentages) reported in minutes per week, MET-minutes per week, METs per week, and percentage of time per day. CRF, WC, and SBP were also extracted.

### Study Risk of Bias Assessment

The Cochrane Collaboration Tool was used for RCTs to assess the risk of bias in individual studies [34]. Risk of bias summary in RCTs was assessed using Review Manager 5.4.1 (The Cochrane Collaboration). Each criterion was rated as low risk, high risk, and unclear risk [29]. Additionally, the global risk of bias was assigned to each study based on the sum of 6 criteria (low risk=0 points, unclear risk=1 point, and high risk=2 points). The global risk of each study ranged from 0 (the lowest) to 12 (the highest). For non-RCTs, the Cochrane Effective Practice and Organization of Care review group methods was used to assess potential bias in individual studies [35]. Another source of bias was whether MVPA measurement methods were objective or subjective. Self-reported data and directly measured physical activity data differed greatly [36]. Tianzhuo Y performed the risk of bias assessment, which was independently verified by XZ. Disagreements between reviewers were resolved through discussion with FL.

### Effect Measures

Only studies reporting MVPA in minutes per week or MET-minutes per week and presenting data as the mean (SD) or mean difference and SD of the difference were included in the meta-analysis. Posttest difference between the intervention group and control group was used as the effect size estimate for data presented as mean (SD), while the pre-post change between the 2 groups was used as the effect size estimate for data presented as mean difference and SD of the difference.

### Synthesis Methods

All statistical analyses were performed using Stata BE, version 17 (StataCorp LLC). SMD (Cohen *d* and 95% CI) was used to calculate the effect sizes for data on continuous variables between the 2 groups. Considering the heterogeneity of different studies, the random-effect model (DerSimonian and Laird method) was used to calculate the weighted effect size. When a single study existed with more than 1 intervention or control group, they were combined to create a single intervention and control group for each study in the meta-analysis [31].

### Subgroup Analysis

Priori-determined subgroup analyses were performed to test for differences in sample size (<50 participants vs ≥50 participants), measurement method (self-reported vs objective), intervention component (single component vs multicomponent), intervention characteristic (standardized vs tailored), intervention duration (<12 weeks vs ≥12 weeks), interaction with health care professionals (interaction vs no interaction), and control characteristic (supervised vs unsupervised). According to the Cochrane Manual, if the number of studies included in this result is less than 10, it is not meaningful and subgroup analysis is not performed [37]. If a subgroup contained only 1 study, the subgroup analysis was not performed for this outcome. In addition, to prevent some studies from using multiple intervention delivery methods, causing some factors such as mutual confounding or possible collinearity, and thus leading to incorrect conclusions in subgroup analysis, we conducted a post hoc subgroup analysis of the intervention delivery methods (wearable-based vs web-based [web portal or smartphone app] vs communication-based [emails, text messages, or telephone counseling]) and did not test the overall statistics or heterogeneity between the groups.

### Publication Bias

Publication bias was assessed visually by a funnel plot and statistically by the Egger test. If the funnel plot or Egger test indicated publication bias, the trim-and-fill (Duval and Tweedie) procedure was used to estimate the pooled effect sizes adjusting for publication bias.

### Sensitivity Analysis

If the *I*^2^ statistic with values showed heterogeneity, a Galbraith plot was made to determine which study was the source of heterogeneity. A sensitivity analysis was performed by removing the single study method. Post hoc sensitivity analyses of the primary outcomes were performed using the fixed-effects model, Hedge test, or excluding non-RCTs. We also examined the effect of individual studies by using the “metan inf” command on the pooled effect size.

### Certainty Assessment

The quality of the evidence was assessed using the Grading of Recommendations Assessment, Development and Evaluation (GRADEpro GDT, web-based version) professional guideline development tool. Although evidence based on RCTs begins as high-quality evidence, to reduce the decline of confidence in the evidence, the quality of evidence was still determined by assessing the (1) risk of bias, (2) inconsistency, (3) indirectness, (4) imprecision, and (5) publication bias [38]. In addition, heterogeneity was assessed using the *I*^2^ statistic with values >75%, and *P*<.10 was used to indicate considerable heterogeneity across studies [39].

## Results

### Selection of the Studies

**Figure 1:**
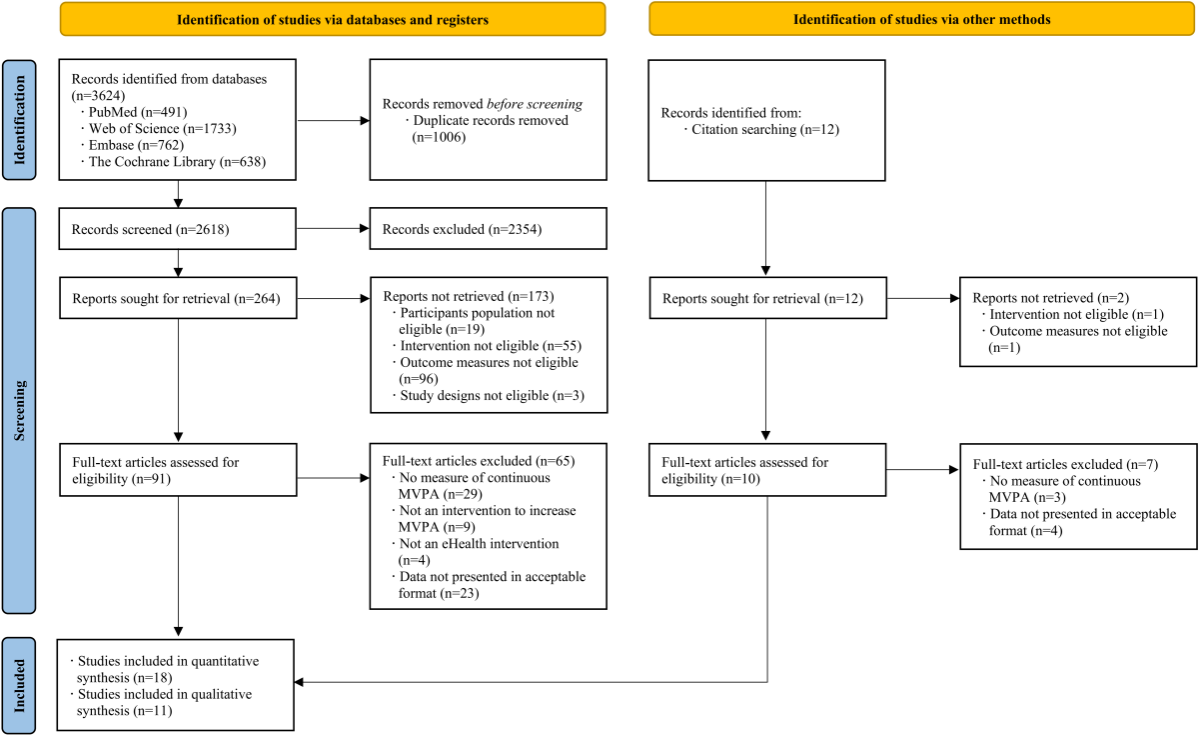
Flowchart for study identification, screening, eligibility, and inclusion. MVPA: moderate-to-vigorous intensity physical activity.

The database search completed on November 27, 2022, identified 3624 records. Four databases were searched: PubMed (n=491), Web of Science (n=1733), Embase (n=762), and Cochrane Library (n=638). Twelve additional records were identified by manually searching reference lists of the included studies and relevant systematic reviews and meta-analyses. After removing duplicates, a total of 2630 records remained. After title and abstract screening, 101 full-text papers were reviewed for eligibility. Twenty-nine studies met the inclusion criteria and were included in the final analysis. Of the 29 studies in this review, 18 were included in the meta-analysis. Cardiovascular-related outcomes were reported in several studies: CRF (n=8), WC (n=5), and SBP (n=10). Figure 1 provides reasons for the exclusion of studies from this review.

### Characteristics of the Studies

The characteristics of the studies are shown in Multimedia Appendix 4. MVPA outcomes are provided in Multimedia Appendix 5. Studies included in the systematic review were published between 2009 and 2022 and conducted in 16 countries (United States of America [n=9], Canada [n=5], Belgium [n=3], Finland, Netherlands, Ireland, New Zealand, Jordan, Norway, United Kingdom, France, Spain, Portugal, Australia, and China). Twenty-three RCTs [40-62], 2 quasi-experimental [63,64], and 4 pre-post [65-68] studies examined changes in MVPA levels following eHealth interventions. All papers were published in English. A total of 3261 patients (mean age range, 54-71 years) in CR participating in 29 studies were included in this systematic review. The sample sizes ranged from 10 [68] to 500 [57]. Most patients in phase II or III CR were referred because of a diagnosis of coronary heart disease, myocardial infarction, coronary artery bypass surgery, coronary artery disease, stable angina, acute coronary syndrome, percutaneous coronary intervention, heart valve repair, percutaneous transluminal coronary angioplasty, heart failure, and heart transplant.

### Intervention Details

Interventions varied widely between and among the studies. Seventeen studies used multiple eHealth components [40,43-45,47,48,50-53,56,57,60,61,63,67,68], and 12 studies used a single eHealth component [41,42,46,49,54,55,58,59, 62,64-66]. Fourteen studies used wearable devices such as heart rate monitors to monitor heart rate or electrocardiography to adjust exercise prescription to reach the target heart rate zone for maintaining optimal levels of physical activity and cardiovascular health [40,43,45,48,60], pedometers or accelerometers to monitor and record physical activity remotely [47,51,52,57,63,64,67,68], and a VTAP device to remind and remain physically active [46]. Twelve studies used web-based portals, with interventions including websites with CVD self-management information about healthy lifestyle behaviors (eg, progressing physical activity incrementally, nutrition, emotion management, smoking cessation) [42-44,48,55,58,62,65], websites with physical activity tracking tools (eg, uploading physical activity data, web-based exercise diaries) for personally tailored physical activity goal setting and feedback on achieving physical activity goals [40,45,53,59], and websites with a web-based discussion forum for communication with CR professionals or nurses and patients for social support [42,43,62]. Nine studies used smartphone apps, the same as web portal, to allow people to set physical activity goals [45,56,67,68], offer exercise sessions [55,56,61,68], and upload physical activity data facilitating review and self-monitoring [45,47,48,56,60,61,68]. Ten studies used messaging services as reminders to carry on with the physical activity programs as indicated in the prescription and support to achieve the physical activity goals [45,47,50,51,56,57,63,66], as feedback according to physical activity performance [51,53,57,60]. Eight studies conducted consultations to obtain tailored feedback on engagement in physical activity via telephone calls [41,44,47,49,50,52,54,60]. Significant non-eHealth aspects of the interventions included the provision of exercise prescriptions [41], verbal or written advice about maintaining physical activity [43,47-50,52-55,60,63,64], and supervised exercise sessions by exercise physicians in outpatients or CR clinics [40,42,44-46,51,60-62]. Few of the interventions were designed using health behavior theories such as the behavior change theory [67,68], Lorig chronic disease self-management model [49], Hibbard patient activation theory [49], Bandura conceptualization of self-efficacy [49], social cognitive theory [44,50], self-efficacy theory [50], planned behavior theory [50], goal-setting theory [57], health action process approach model [56,59], and self-determination theory [58].

### Risk of Bias in Studies

**Figure 2:**
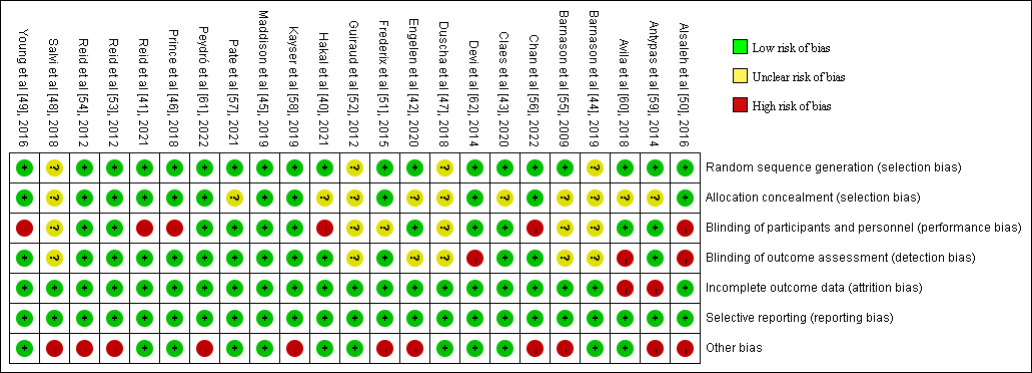
Risk of bias summary in randomized controlled trials [40-62].

**Figure 3:**
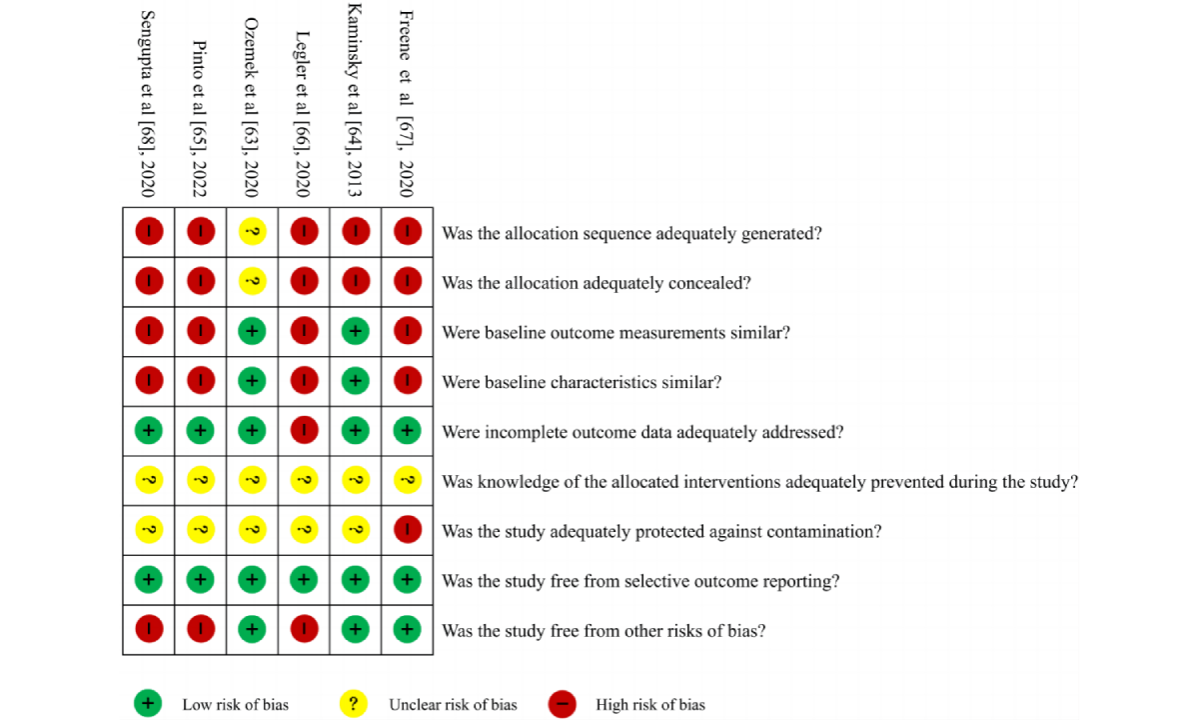
Risk of bias summary in nonrandomized controlled trials [63-68].

Figure 2 shows the assessment of risk of bias in RCTs [40-62] (n=23). The greatest risk of bias resulted from “other bias” because self-reported methods were largely used for the measurement of MVPA [42,48,50,51,53-56,58,59,61] (11/23, 48%). Second, the risk of bias came from blinding participants and personnel [40,41,46,49,50,56] (6/23, 26%). Nevertheless, due to the nature of the physical activity intervention, it was impossible to blind patients or the health care professionals delivering the intervention to group allocation. Three (13%) studies [50,60,62] had a high risk of blinding outcome assessment for not being blinded to outcome evaluators. Only 2 (9%) studies [59,60] had a high risk of incomplete outcome data; in 1 study [59], the number and cause of missing outcome data were inconsistent between groups, and the other study [60] had more missing data on physical activity outcome, which may have affected the effectiveness of the intervention. There was no high-risk bias in random sequence generation, allocation concealment, or selective reporting. The global risk of bias scores ranged from 0 to 6, with a mean score of 3.09. Figure 3 shows the risk of bias summary for non-RCTs [63-68], which is drawn with reference to the Review Manager 5.4.1 risk of bias assessment. Because these 6 studies were non-RCTs, the sequence generation and allocation were high risks or unclear risks. Four of these were pre-post studies [65-68], and there was no baseline comparison between groups; therefore, both baseline outcome measurements and characteristics were high risk. One study [67] did not blind outcome evaluators; therefore, there was a high risk of preventing contamination. Three studies [65,66,68] assessed MVPA by using the self-reported method; therefore, there was a high risk in terms of other risks of bias.

### Results of Individual Studies and Results of Syntheses

#### Effect on MVPA

**Figure 4:**
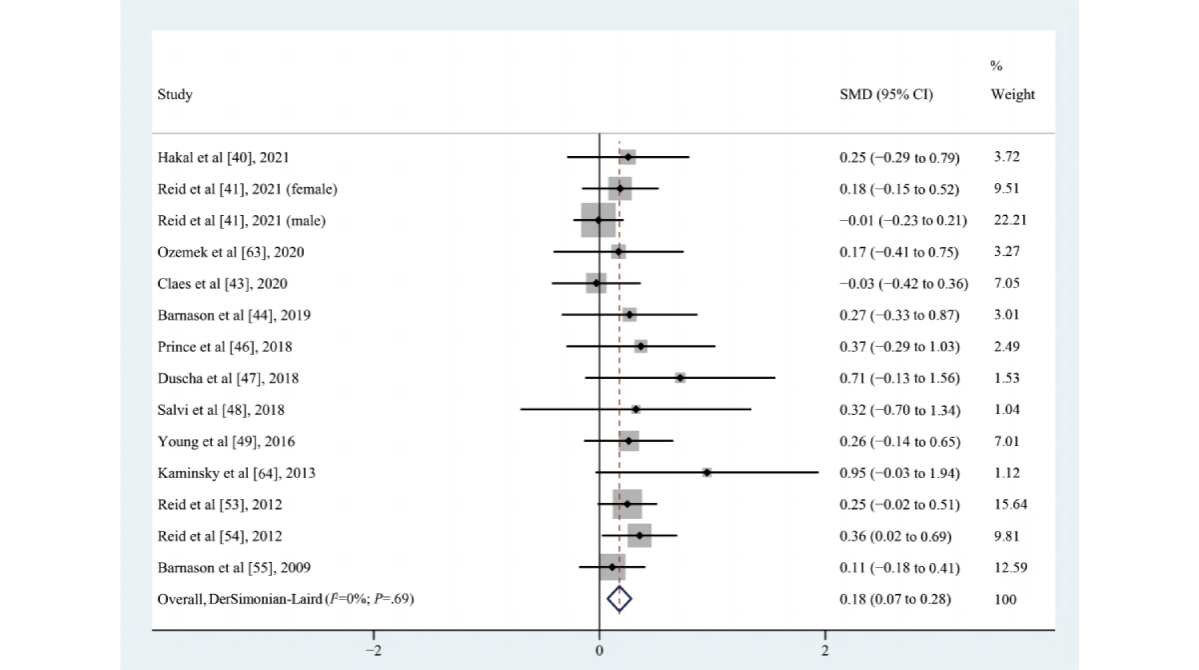
Effects of eHealth on changes in the time spent on moderate-to-vigorous intensity physical activity among patients in cardiac rehabilitation [40,41,43,44,46-49,53-55,63,64]. SMD: standardized mean difference.

A summary of the primary outcomes by study design in the included studies is shown in Multimedia Appendix 6. Of the 29 included studies [41-68], 18 [40,41,43,44,46-49,53-55, 57,58,60,63-65,67] assessed the impact of eHealth intervention on the time spent on MVPA and reported mixed results. Of these 18 studies, 14 [40,41,43,44,46-49,53-55,57,58,60] were RCTs, 2 [63,64] were quasi-experimental studies, and 2 [65,67] were pretest/posttest studies. Four studies [43,47,55,57] reported that the eHealth intervention exerted a positive effect on the time spent on MVPA, 2 [64,65] reported that the pre-post difference between groups was statistically significant, 4 (ie, [41] for the male cohort, [44,49,63]) reported that no difference existed between the intervention and control groups for the time spent on MVPA, and 5 (ie, [40], [41] for the female cohort, [46,58,60]) suggested a trend toward an increase in the time spent on MVPA after the eHealth intervention, although the difference was not statistically significant. According to the requirements for inclusion in the meta-analysis, 13 [40,41,43,44,46-49,53-55,63,64] eligible studies (n=1430) were meta-analyzed, and the results demonstrated that eHealth interventions increased the time spent on MVPA, although the effect size was small (SMD=0.18, 95% CI 0.07-0.28; *P*=.001; Q(13)=10; *P*=.69; *I*^2^=0%; as shown in Figure 4).

#### Effect on MPA

**Figure 5:**
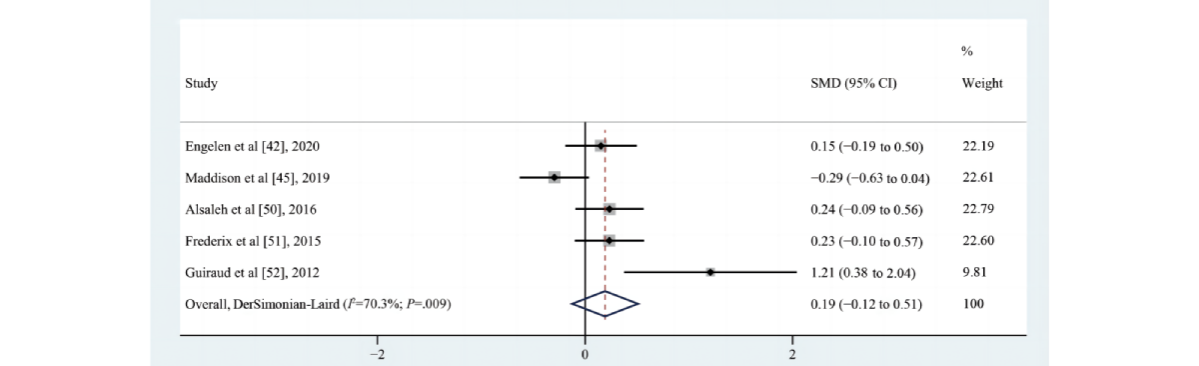
Effects of eHealth on changes in the time spent on moderate-intensity physical activity among patients in cardiac rehabilitation [42,45,50-52]. SMD: standardized mean difference.

Thirteen studies [42,45,50-52,56,59-62,65,66,68] assessed the impact of the eHealth intervention on the time spent on MPA; 10 were RCTs [42,45,50-52,56,59-62] and 3 were pre-post studies [65,66,68]. Four [50,52,61,62] reported a positive effect, 2 [45,66] reported that no difference existed between groups, and 4 [42,59,60,68] suggested a trend toward an increase, although not statistically significant. According to the requirements for inclusion in the meta-analysis, 5 [42,45,50-52] eligible studies (n=594) were meta-analyzed, and the results of minutes per week of MPA were not statistically significant. There were no changes in the time spent on MPA (SMD=0.19, 95% CI –0.12 to 0.51; *P*=.23; Q(4)=13.49; *P*=.009; *I*^2^=70.3%), as shown in Figure 5.

#### Effect on VPA

**Figure 6:**
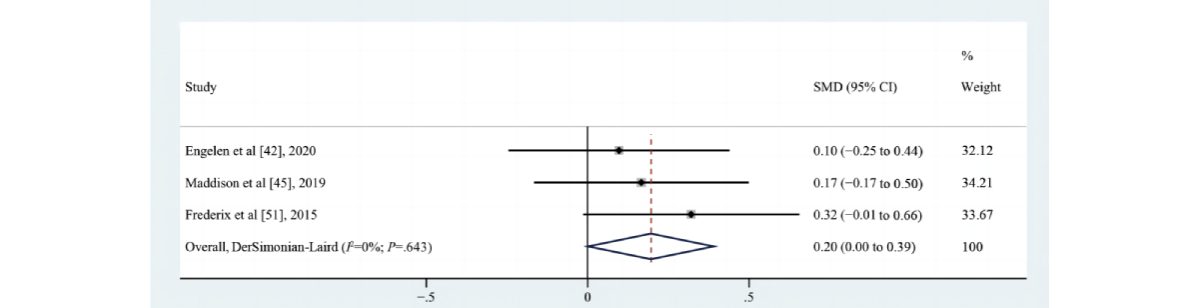
Effects of eHealth on changes in the time spent on vigorous-intensity physical activity among patients in cardiac rehabilitation [42,45,51]. SMD: standardized mean difference.

Eight studies [42,45,51,56,59-61,65] assessed the impact of eHealth interventions on the time spent on vigorous-intensity physical activity (VPA); 7 [42,45,51,56,59-61] were RCTs, and 1 study [65] had a pre-post design. Four [42,45,59,60] of them suggested a trend toward an increase, but the increase was not statistically significant; only 1 study [65] showed statistically significant differences between pre-post results within the group. According to the requirements for inclusion in the meta-analysis, 3 eligible studies [42,45,51] (n=424) were meta-analyzed, and the results demonstrated that eHealth interventions increased the time spent on VPA (SMD=0.20, 95% CI 0.00-0.39; *P*=.048; Q(2)=0.88; *P*=.64; *I*^2^=0%), as shown in Figure 6.

#### Effect on CRF

**Figure 7:**
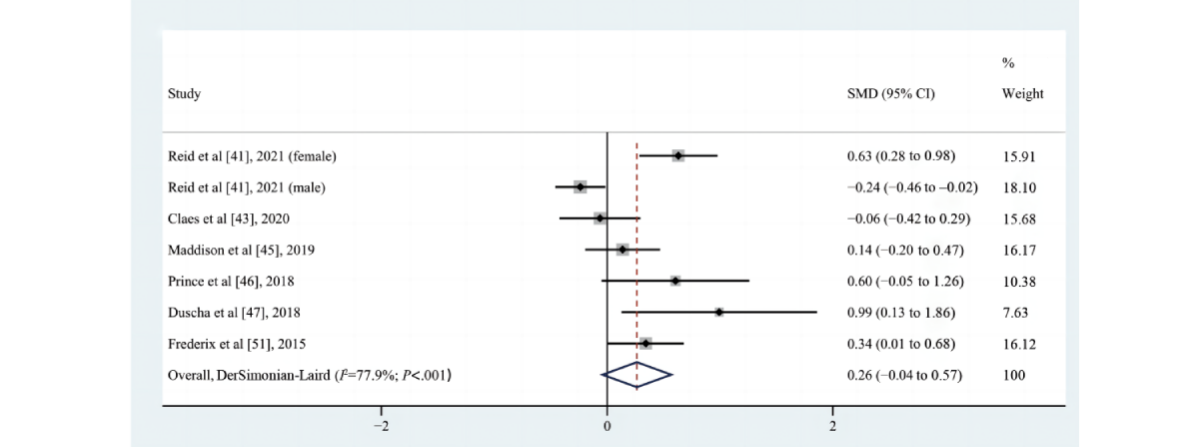
Effects of eHealth on changes in cardiorespiratory fitness among patients in cardiac rehabilitation [41,43,45-47,51]. SMD: standardized mean difference.

A summary of the secondary outcomes by study design in the included studies is shown in Multimedia Appendix 6. Of the 8 studies [41,43,45-47,51,60,67] that assessed the impact of eHealth intervention on changes in CRF, 2 [51,60] reported a positive effect, 1 [67] reported a statistically significant pre-post difference between groups, 2 (ie, [41] for the male cohort, [43]) reported that there was no difference between groups, and 4 ([41] for the female cohort, [45-47]) suggested a trend of increase, although not statistically significant. One of the studies [67] used the 6-minute walk test to assess CRF, and 7 studies [41,43,45-47,51,60] expressed CRF as peak oxygen uptake. A meta-analysis of 6 [41,43,45-47,51] pooled studies (n=911) was not statistically significant (SMD=0.26, 95% CI –0.04 to 0.57; *P*=.09; Q(6)=27.14; *P*<.001; *I*^2^=77.9%, as shown in Figure 7). Consistent with the results of the meta-analysis, the study by Claes et al [43] also found that this effect on MVPA did not translate into a positive effect on CRF, indicating that this effect might not be large enough to be clinically important. However, it was found that 4 studies (ie, [41] for the female cohort, [46,47,51]) (4/7, 57%) included in the CRF meta-analysis reported an increasing trend in the time spent on MVPA and the peak oxygen uptake simultaneously; this increase in the duration of higher intensity physical activity likely accounted for their preserved peak oxygen uptake [47].

#### Effect on WC

**Figure 8:**
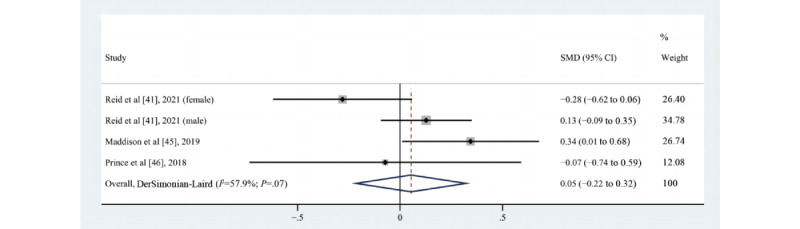
Effects of eHealth on changes in waist circumference among patients in cardiac rehabilitation [41,45,46]. SMD: standardized mean difference.

Of the 5 studies [41,45,46,60,67] that assessed the impact of eHealth intervention on changes in WC, 2 ([41] for the male cohort, [45]) reported no statistically significant differences between groups, and 4 ([41] for the female cohort, [46,60,67]) suggested a trend toward improvement, although there was no statistically significant difference. A meta-analysis of 3 pooled studies [41,45,46] (n=625) was not statistically significant (SMD=0.05, 95% CI –0.22 to 0.32; *P*=.69; Q(3)=7.13; *P*=.07; *I*^2^=57.9%, as shown in Figure 8). The study by Reid et al [41] found a trend toward a lower reduction in WC in women but a negative impact on WC in men, which might have been spurious, or perhaps men might have eaten more as they were partaking in a physical activity intervention.

#### Effect on SBP

**Figure 9:**
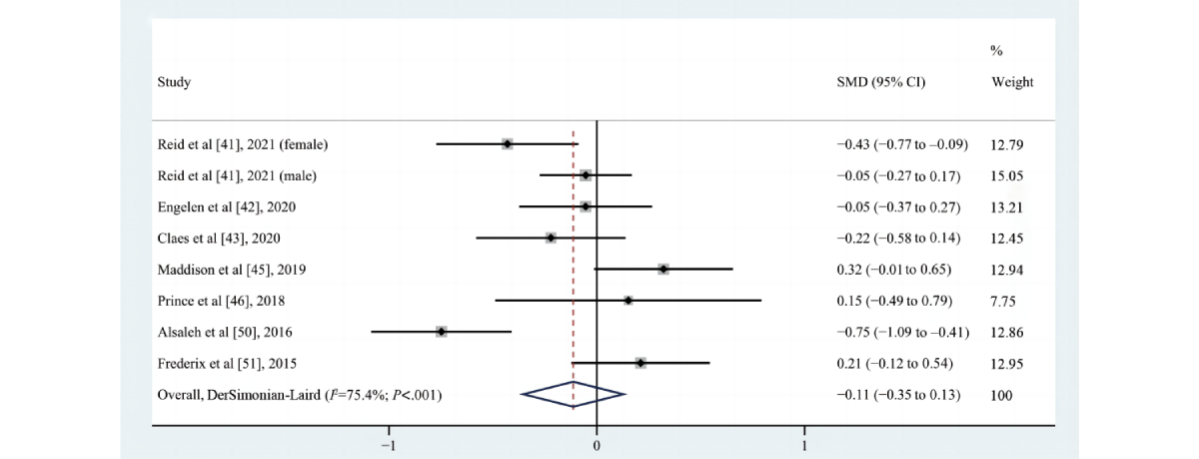
Effects of eHealth on changes in systolic blood pressure among patients in cardiac rehabilitation [41-43,45,46,50,51]. SMD: standardized mean difference.

Of the 10 studies [41-43,45,46,50,51,60,62,67] that assessed the impact of eHealth intervention on changes in SBP, 2 [50,62] reported a statistically significant difference between groups, but one of the studies [62] reported a negative effect, 1 [67] reported that the pre-post difference between groups was statistically significant, 4 [45,46,51,60] reported that there was no difference between the intervention and the control groups, and 3 [41-43] suggested there was a trend toward improvement, but the improvement was not statistically significant. The result of reporting negative effects was unexpected, with statistically significantly reduced SBP in the control group compared to that in the web-based CR group, but Devi et al [62] did not explain this result. A meta-analysis of 7 pooled studies [41-43,45,46,50,51] (n=1183) was not statistically significant (SMD=–0.11, 95% CI –0.35 to 0.13; *P*=.36; Q(7)=28.4; *P*<.001; *I*^2^=75.4%), as shown in Figure 9.

### Subgroup Analyses

Because only the number of studies that included MVPA outcomes exceeded 10, only subgroup analyses of MVPA were conducted. Previously determined subgroup analyses included sample size, measurement method, intervention component, intervention characteristic, intervention duration, interaction with health care professionals, and control group characteristics. The results of the subgroup analyses identified no statistically significant changes in weekly minutes of MVPA (Multimedia Appendix 7). Post hoc determined subgroup analyses of MVPA were intervention delivery methods (wearable-based vs web-based vs communication-based). Because 7 studies [40,43,44,47,48,53,63] used both or 3 intervention delivery methods and to avoid some factors as confounding factors, we did not test the overall statistic or the heterogeneity between groups. Multimedia Appendix 8 shows the post hoc determined subgroup analyses of MVPA. We found statistically significant increases in the time spent on MVPA among patients in CR in studies that incorporated 3 delivery methods, namely, wearable-based interventions, web-based interventions, and communication-based interventions (Multimedia Appendix 9).

### Publication Bias

Because drawing a funnel plot requires at least 10 original studies, we performed the funnel plot analysis only for the MVPA outcomes. Multimedia Appendix 10 shows the funnel plot for the MVPA outcomes in 13 studies, which indicate the presence of a funnel plot asymmetry. The *P* value of Egger test was statistically significant (*P*=.01), suggesting publication bias in the MVPA outcome.

### Sensitivity Analyses

The Galbraith plot was drawn, and sensitivity analysis was performed using the method of removing a single study to find the cause of heterogeneity in MPA outcomes. Through the Galbraith plot, we found that the study by Guiraud et al [52] intersects with a 95% CI regression line, suggesting that it might be the anomaly of the source of heterogeneity (Multimedia Appendix 11). After the removal of the study by Guiraud et al [52], the MPA outcome was still not statistically significant (SMD=0.08, 95% CI –0.17 to 0.33; *P*=.52), but there was a statistically significant decrease in heterogeneity (Q(3)=6.71; *P*=.08; *I*^2^=55.3%). Because of the publication bias in MVPA outcomes, we used the “meta trim fill” command to estimate the pooled effect size adjusted for publication bias, which found inconsistency with the results before adjustment. The adjusted pooled effect size was not statistically significant, indicating that the publication bias was large and that the results were not stable. Multimedia Appendix 12 shows the post hoc sensitivity analyses. We found changes in the MPA outcome after removing 1 study [45] with a greater influence on the pooled effect size (SMD=0.30, 95% CI 0.03-0.56; *P*=.03; Q(3)=5.41; *P*=.14; *I*^2^=44.6%). We also found similar changes in the CRF outcome; after removing 1 study (ie, [41] for the male cohort) with a greater influence on the pooled effect size, the results were statistically significant (SMD=0.35, 95% CI 0.09-0.62; *P*=.009; Q(5)=11.76; *P*=.04; *I*^2^=57.5%).

### Certainty of Evidence

The quality of evidence was assessed using GRADEpro GDT. The evidence rating began as “high” given that the majority of included studies were RCTs (16/18, 89%). Most information from studies was at low or unclear risk of bias. Although 26% (6/23) of the studies did not blind or report blinding of the participants, this was typically not possible in physical activity interventions due to their nature. Additionally, 48% (11/23) of the studies reported using a measurement of physical activity that was self-reported, and our subgroup analyses reported no statistically significant difference between objective and self-reported physical activity. In addition to the lack of blindness of participants and the risk of bias caused by self-reported measurements, the potential limitations of other unclear and high risks were unlikely to reduce confidence in the effect estimates. Therefore, we did not downgrade the quality of evidence for the risk of bias. Inconsistency was assessed by considering the heterogeneity of the studies, and an *I*^2^>75% and *P* value <.01 were considered as cutoffs for considerable heterogeneity. Our meta-analysis identified a substantial degree of inconsistency across studies in the MPA outcome (*I*^2^=70.3% and *P*=.009, as shown in Figure 5). However, only the *P* value was less than .10, and the *I*^2^ statistic did not exceed 75%; therefore, we only downgraded one level of the quality of the evidence from high to moderate due to the heterogeneity. The evidence was not downgraded for indirectness or imprecision in the MVPA and VPA outcomes, as the pooled sample size was relatively large and the 95% CI was not wide. However, the 95% CI of the pooled effect size of the MPA outcome contained an invalid value; therefore, we downgraded the quality of evidence from moderate to low due to imprecision. Finally, a funnel plot and Egger test detected publication bias in the MVPA outcome in 13 studies. The funnel plot showed asymmetry, with a statistically significant *P* value in the Egger test at .01. Therefore, the quality of the evidence for publication bias was downgraded from high to moderate. The quality of evidence used in our meta-analysis for the MVPA outcome was determined to be moderate, the MPA outcome was determined to be low, and the VPA outcome was determined to be high. The MVPA for moderate quality of evidence and the VPA for high quality of evidence provide reasonable confidence in the estimate of effect. The overall certainty of the secondary outcomes evidence ranged from very low to low because the pooled effect sizes contained an invalid value and the heterogeneity was high (Multimedia Appendix 13). However, it also suggests that further research is highly likely to change the results.

## Discussion

### Principal Findings

To our knowledge, this is the first systematic review and meta-analysis to examine the effectiveness of eHealth interventions in improving MVPA and cardiovascular-related outcomes in patients in CR. We found that eHealth interventions statistically significantly increased MVPA and VPA, as measured by minutes per week. However, there was no statistically significant benefit of eHealth interventions for MPA and cardiovascular-related outcomes, including CRF, WC, or SBP, although improvements were seen in pooled analyses. In prior determined subgroup analyses, we found no subgroup that produced intergroup differences. In post hoc determined subgroup analysis by eHealth intervention delivery, there was a particular benefit seen for wearable-based, web-based, and communication-based intervention approaches.

### Effects of the eHealth Interventions on the Primary Outcomes

eHealth interventions could offer a level of impact, accessibility, affordability, cost savings, and benefits to people, which would not have been possible with conventional CR [69]. The study of dose-response relationship between physical activity and all-cause, cardiovascular, or cancer mortality by Arem et al [70] found a 20% lower mortality risk among patients who performed less than the recommended minimum of MVPA (75 minutes per week of VPA or 150 minutes per week of MPA), a 31% lower risk at 1-2 times the recommended minimum, and a 37% lower risk at 2-3 times the minimum. Our review is the first to focus on patients in CR and to definitively demonstrate that eHealth interventions effectively improve the minutes per week of engagement in MVPA. Although the effect size of our meta-analysis was small, it also indicated that eHealth interventions compared with non-eHealth interventions can increase MVPA minutes per week in patients in phase II or III CR, which might have important effects on the health of people with CVD and reduce cardiovascular mortality. The results of the time spent on MVPA minutes per week by Patterson et al [23] were consistent with those reported in our review, and the pooled effect size of the meta-analysis was large (SMD=40.35, 95% CI 1.03-79.67; *P*=.04), but the sample size of the included studies was small (n=600). Moreover, there was moderate heterogeneity (*I*^2^=51%; *P*=.06) and limited representation of each CVD diagnostic group (such as peripheral arterial disease, stroke, and hypertension); therefore, the results should be interpreted with caution. The improvement in MVPA minutes per week found in our meta-analysis was slightly lower than that found in a meta-analysis of 4 studies by Su et al [71], who evaluated the effects of an eHealth CR intervention on the physical activity in a population with coronary heart disease (SMD=0.24, 95% CI 0.04-0.44; *P*=.02). The slightly lower increase in MVPA in our study could perhaps be explained by the fact that Su et al [71] did not distinguish between the intensities of physical activity and that MVPA was precisely lacking in patients in CR. We obtained the same results in weekly minutes of VPA, showing that eHealth interventions can increase the time spent on VPA in patients in CR. However, the result of changes in the weekly minutes of MPA was not statistically significant, and the possible reason was that this review examined 1 study with large impacts on the pooled effect size. When this study was removed using the “metan inf” command and sensitivity analysis, the result of the meta-analysis of the MPA outcome was statistically significant. The removed study was that by Maddison et al [45] comparing the effectiveness of a remotely monitored exercise-based CR program with that of a center-based program in patients undergoing phase II CR. The remotely monitored exercise-based program was no more effective than the center-based program for MPA, with the possible explanation being that center-based, supervised, and face-to-face exercise improved access, uptake, and adherence to CR for these patients who had recently been discharged from hospital and needed to participate in phase II CR.

### Effects of the eHealth Interventions on the Secondary Outcomes

Increased MVPA combined with decreased sedentary behavior can positively change CRF and improve clustered cardiometabolic risk (eg, WC, SBP) by mediating CRF [72]. We observed a nonstatistical significant increase in CRF—an important clinical predictor of the future risk of readmissions for CVD and all-cause mortality [73]. However, when the “metan inf” command and the sensitivity analysis were used to remove a study that had a large impact on the pooled effect size, the meta-analysis result of the CRF outcome was statistically significant. The removed study was the male participants in the study by Reid et al [41] among the phase III patients in CR, stratified by gender, using the eHealth intervention approach of telephone counseling. The explanation for the finding that the telephone counseling eHealth intervention might not be effective for male participants was because they already had higher MVPA levels and better CRF at the completion of phase II CR. WC as a strong predictor of coronary heart disease risk may be better than body mass index among men and women 60 years of age and older [74]. Nevertheless, we observed no statistically significant difference in WC. Although CR is a multidisciplinary intervention, weight loss programs have traditionally not been included [75], which may also explain why our meta-analysis of the WC outcome was not statistically significant. The eHealth interventions did not focus on dietary advice and weight management in any of the 3 studies we included. We also observed no statistically significant difference in SBP, which was the same as the result of the meta-analysis by Widmer et al [16] for digital health interventions for the secondary prevention of CVD. This may be explained by the fact that exercise-induced physiological adaptations are usually dose-dependent [29], and because the studies included in the meta-analysis selected and implemented different eHealth intervention techniques and exercise prescriptions with varying levels of appeal to patients and patient engagement, the eHealth interventions may not have provided the sufficient intensity of intervention to achieve improvements in SBP.

### Discussion of Subgroup Analyses

Subgroup analyses showed no differences in MVPA with any of the priori subgroup comparisons. Regarding sample size, these findings are in agreement with those of a meta-analysis reporting the use of smartphone apps to increase MVPA [23]. Our subgroup analysis did not find an effect of self-reported or objective measurement methods on MVPA. Nevertheless, a systematic review by Prince et al [36] found that self-reported measures of physical activity were all higher or lower than directly measured physical activity levels. We also grouped by type of control group to differentiate effects between actively and passively controlled studies and found no difference between the subgroups. However, in contrast to our results, Rawstorn et al [76] demonstrated that the lack of supervision in the control group had an impact on the physical activity level, that is, the physical activity level was significantly higher following supervised CR (eg, center-based or outpatient CR) than unsupervised CR (eg, usual care). Our findings also showed no difference in MVPA between the number of intervention components, tailored eHealth intervention, length of the intervention duration, and their interaction with health care professionals. In addition, for the post hoc subgroup analysis of intervention delivery methods, all 3 main intervention delivery methods (ie, wearable-based vs web-based vs communication-based) were statistically significant and showed effectiveness. These findings are informative for the provision of rehabilitation during the COVID-19 pandemic. Social isolation during the COVID-19 pandemic could have increased the physical inactivity and the global burden of CVD [77]. Moreover, the COVID-19 pandemic led to the closure of many CR centers, resulting in many eligible people being unable to participate in the optimization of secondary prevention and physical performance [78]. This calls for action on cardiac telerehabilitation [78], and the results of our subgroup analyses demonstrate the potential of eHealth interventions. Cardiac telerehabilitation teams and patients can choose the best personalized tailored eHealth intervention delivery to improve the time spent on MVPA based on convenience, accessibility, affordability, and cost-effectiveness.

### eHealth Interventions and Adherence

eHealth interventions as novel approaches can reduce dropout rates and increase availability in CR [79]; therefore, we additionally discussed the impact of eHealth interventions on intervention compliance and physical activity adherence in patients in CR. Of the 29 studies included in the systematic review, 14 [40,41,43,45,48-50,52-54,59,62,65,67] focused on adherence to the intervention. Multimedia Appendix 14 shows the definition, measures, and findings of adherence to eHealth interventions. Most studies had documented adherence to CR programs through the completion of exercise training sessions [41,43,45,48,65], participation in other scheduled sessions [43,53,54,62,65,67], website log-ins [40,59], and interactions with health care professionals by patients in CR [40,53]. The sessions were delivered through eHealth technologies; log-ins to the website were automatically recorded, and interactions with health care professionals were recorded in terms of the number of text messages and emails sent. Therefore, the adherence to eHealth interventions was measured and evaluated objectively. Fewer studies have focused directly on adherence to physical activity. Adherence was assessed either by the number of minutes of exercise actually performed by patients in CR as a percentage of the prescribed minutes of exercise [48] by comparing the percentage of people in the intervention and control groups who achieved 80% of the recommended 150 minutes per week of moderate or higher intensity physical activity in each group [49], by comparing longitudinally the completion of physical activity diaries [50], or by comparing the percentage change from baseline to the end of follow-up in the number of people who achieved different minutes of MPA [52]. Although the methods used to assess adherence were varied, 6 studies clearly affirmed in the text that the eHealth interventions adopted improved adherence to physical activity [43,49,52,59] or that patients in CR had high adherence to eHealth interventions aimed at increasing physical activity [48,50]. This also confirmed that eHealth technology–based interventions are indeed powerful initiatives to promote intervention compliance and physical activity adherence. The results about adherence varied depending on the choice of indicators [48], which may explain why the other 8 studies did not report improvements in adherence, as the interpretation of adherence is limited by the wide variation in the methodologies used and the definition of adherence [65].

### Limitations

#### Limitations of the Evidences Included in This Review

The evidence included in this review has 4 limitations. First, as shown in our evaluation of the risk of bias, 13 studies (13/18, 72%) included in the meta-analysis did not blind or report blinding of participants and personnel. Due to the nature of physical activity interventions, blinding was not typically possible and, therefore, should not be considered as a reason for downgrading the quality of evidence. Beyond performance bias, there is an explicit research limitation, with nearly half of the studies (14/29, 48%) measuring physical activity data based on self-reported methods, which may greatly reduce the validity of the data. Nevertheless, our subgroup analysis showed no significant statistical differences between objective and self-reported measures. Therefore, again, this cannot be used as a reason to reduce the quality of evidence. Second, the meta-analysis results of MPA, CRF, WC, and SBP showed high heterogeneity, suggesting that the results of the studies should be interpreted and generalized with caution. At the same time, the quality evaluation showed low evidence but the low quality of evidence precisely showed that further research could change the results of our meta-analysis. eHealth interventions had great potential for improving MPA, CRF, WC, and SBP. Third, we found a large publication bias in the meta-analysis results of MVPA, and the results were found to be unstable through sensitivity analysis. However, the “meta trim fill” command adds several nonexistent small sample studies according to the symmetry principle. It calculates the pooled effect size on this basis, which is controversial. Therefore, publication bias of the MVPA outcome should be treated with caution. Finally, as much as we try to avoid it, some key references may have been missed due to search strategies and criteria and the exclusion of grey literature.

#### Limitations in the Review Processes Used

There are also 2 limitations in the review process used. First, the focus of our study was on the effect of eHealth-based CR on minutes of MVPA per week rather than its effect on the clinical end points such as death and other clinical events. This is probably the major limitation of our study. One of the reasons our study did not focus on its effect on the clinical end points is that relevant meta-analyses have been performed to investigate clinical end points. In a meta-analysis to determine whether telehealth interventions provide effective secondary prevention compared with CR or compared with usual care, Jin et al [80] found that telehealth interventions significantly reduced the risk of rehospitalization or cardiac events over a 6-36 month period (risk ratio=0.56, 95% CI 0.39-0.81; *P*<.001). Another reason is that MVPA has been shown to be inversely associated with all-cause mortality and cardiovascular mortality [33]. A meta-analysis by Hupin et al [81] found that in adults aged ≥ 60 years, even a low dose of MVPA reduced mortality by 22%. Therefore, our study focused on whether eHealth-based CR could increase the number of minutes of MVPA per week. Second, all outcome indicators used in this study were surrogate parameters. Nevertheless, the primary and secondary outcome indicators we used, including minutes of MVPA per week, CRF, WC, and SBP, are all major risk factors for cardiovascular death and cardiovascular events [25,28,29] and can all be measured using quantitative methods.

### Conclusion

In conclusion, our systematic review and meta-analysis found that eHealth interventions are effective at increasing MVPA and VPA but not MPA or clinically relevant cardiovascular-related outcomes among patients in CR. With the rapid development of digital technology and the COVID-19 pandemic, eHealth interventions provide rich opportunities for CR and cardiovascular-related healthy lifestyle behavior changes. This review confirms the timely use of eHealth approaches to improve MVPA engagement times in a population in CR. Our review mainly focused on people diagnosed with coronary artery disease and referred for CR, but with the incidence increasing year by year, future research is necessary to focus on peripheral artery disease and stroke and determine the long-term effects of eHealth interventions. Our findings will interest physicians, nurses, relevant health care providers, and intervention program makers in CR and encourage them to find more convenient, effective, and affordable ways to increase the MVPA time in patients in CR.

## Appendix

Multimedia Appendix 1 Amendments to the information provided at registration.

Multimedia Appendix 2 PRISMA (Preferred Reporting Items for Systematic Reviews and Meta-Analyses) 2020 checklist.

Multimedia Appendix 3 Search strategy.

Multimedia Appendix 4 Study characteristics.

Multimedia Appendix 5 Moderate-to-vigorous intensity physical activity, moderate-intensity physical activity, and vigorous-intensity physical activity outcomes data from the included studies.

Multimedia Appendix 6 Summary of primary and secondary outcomes in the included studies.

Multimedia Appendix 7 Priori determined subgroup analyses of moderate-to-vigorous intensity physical activity.

Multimedia Appendix 8 Post hoc determined subgroup analyses without overall statistic or heterogeneity between groups of moderate-to-vigorous intensity physical activity.

Multimedia Appendix 9 Post hoc subgroup analyses without overall statistic of intervention delivery methods for the time spent on moderate-to-vigorous intensity physical activity among patients in cardiac rehabilitation.

Multimedia Appendix 10 Funnel plot of effects of eHealth on changes in the time spent on moderate-to-vigorous intensity physical activity among patients in cardiac rehabilitation.

Multimedia Appendix 11 Galbraith plot of the moderate-intensity physical activity outcome.

Multimedia Appendix 12 Sensitivity analyses of the primary and secondary outcomes.

Multimedia Appendix 13 Summary of the quality of the evidence for eHealth interventions versus that for control.

Multimedia Appendix 14 Definitions, measures, and findings of adherence to eHealth interventions.

## Abbreviations

CR: cardiac rehabilitation
CRF: cardiorespiratory fitness
CVD: cardiovascular disease
GRADEpro GDT: Grading of Recommendations Assessment, Development and Evaluation
MET: metabolic equivalents of a task
MPA: moderate-intensity physical activity
MVPA: moderate-to-vigorous intensity physical activity
PRISMA: Preferred Reporting Items for Systematic Reviews and Meta-Analyses
PROSPERO: International Prospective Register of Systematic Reviews
RCT: randomized controlled trial
SBP: systolic blood pressure
SMD: standardized mean difference
VPA: vigorous-intensity physical activity
WC: waist circumference

## References

[1] Cardiovascular diseases (CVDs). World Health Organization.

[2] Roth GA, Johnson C, Abajobir A, Abd-Allah F, Abera SF, Abyu G, Ahmed M, Aksut B, Alam T, Alam K, Alla F, Alvis-Guzman N, Amrock S, Ansari H, Ärnlöv Johan, Asayesh H, Atey TM, Avila-Burgos L, Awasthi A, Banerjee A, Barac A, Bärnighausen Till, Barregard L, Bedi N, Belay Ketema Ezra, Bennett D, Berhe G, Bhutta Z, Bitew S, Carapetis J, Carrero JJ, Malta DC, Castañeda-Orjuela Carlos Andres, Castillo-Rivas J, Catalá-López Ferrán, Choi J, Christensen H, Cirillo M, Cooper L, Criqui M, Cundiff D, Damasceno A, Dandona L, Dandona R, Davletov K, Dharmaratne S, Dorairaj P, Dubey M, Ehrenkranz R, El Sayed Zaki Maysaa, Faraon EJA, Esteghamati A, Farid T, Farvid M, Feigin V, Ding EL, Fowkes G, Gebrehiwot T, Gillum R, Gold A, Gona P, Gupta R, Habtewold TD, Hafezi-Nejad N, Hailu T, Hailu GB, Hankey G, Hassen HY, Abate KH, Havmoeller R, Hay SI, Horino M, Hotez PJ, Jacobsen K, James S, Javanbakht M, Jeemon P, John D, Jonas J, Kalkonde Y, Karimkhani C, Kasaeian A, Khader Y, Khan A, Khang Y, Khera S, Khoja AT, Khubchandani J, Kim D, Kolte D, Kosen S, Krohn KJ, Kumar GA, Kwan GF, Lal DK, Larsson A, Linn S, Lopez A, Lotufo PA, El Razek Hassan Magdy Abd, Malekzadeh R, Mazidi M, Meier T, Meles KG, Mensah G, Meretoja A, Mezgebe H, Miller T, Mirrakhimov E, Mohammed S, Moran AE, Musa KI, Narula J, Neal B, Ngalesoni F, Nguyen G, Obermeyer CM, Owolabi M, Patton G, Pedro J, Qato D, Qorbani M, Rahimi K, Rai RK, Rawaf S, Ribeiro A, Safiri S, Salomon JA, Santos I, Santric Milicevic Milena, Sartorius B, Schutte A, Sepanlou S, Shaikh MA, Shin M, Shishehbor M, Shore H, Silva DAS, Sobngwi E, Stranges S, Swaminathan S, Tabarés-Seisdedos Rafael, Tadele Atnafu Niguse, Tesfay F, Thakur JS, Thrift A, Topor-Madry R, Truelsen T, Tyrovolas S, Ukwaja KN, Uthman O, Vasankari T, Vlassov V, Vollset SE, Wakayo T, Watkins D, Weintraub R, Werdecker A, Westerman R, Wiysonge CS, Wolfe C, Workicho A, Xu G, Yano Y, Yip P, Yonemoto N, Younis M, Yu C, Vos T, Naghavi M, Murray C (2017). Global, Regional, and National Burden of Cardiovascular Diseases for 10 Causes, 1990 to 2015. J Am Coll Cardiol.

[3] Dalal HM, Doherty P, Taylor RS (2015). Cardiac rehabilitation. BMJ.

[4] Fleg JL, Forman DE, Berra K, Bittner V, Blumenthal JA, Chen MA, Cheng S, Kitzman DW, Maurer MS, Rich MW, Shen W, Williams MA, Zieman SJ, American Heart Association Committees on Older PopulationsExercise Cardiac RehabilitationPrevention of the Council on Clinical Cardiology‚ Council on CardiovascularStroke Nursing‚ Council on LifestyleCardiometabolic He (2013). Secondary prevention of atherosclerotic cardiovascular disease in older adults: a scientific statement from the American Heart Association. Circulation.

[5] Cowie A, Buckley J, Doherty P, Furze G, Hayward J, Hinton S, Jones J, Speck L, Dalal H, Mills J, British Association for Cardiovascular PreventionRehabilitation (BACPR) (2019). Standards and core components for cardiovascular disease prevention and rehabilitation. Heart.

[6] Piercy KL, Troiano RP (2018). Physical Activity Guidelines for Americans From the US Department of Health and Human Services. Circ Cardiovasc Qual Outcomes.

[7] Caspersen CJ, Powell KE, Christenson GM (1985). Physical activity, exercise, and physical fitness: definitions and distinctions for health-related research. Public Health Rep.

[8] Bull FC, Al-Ansari SS, Biddle S, Borodulin K, Buman MP, Cardon G, Carty C, Chaput J, Chastin S, Chou R, Dempsey PC, DiPietro L, Ekelund U, Firth J, Friedenreich CM, Garcia L, Gichu M, Jago R, Katzmarzyk PT, Lambert E, Leitzmann M, Milton K, Ortega FB, Ranasinghe C, Stamatakis E, Tiedemann A, Troiano RP, van der Ploeg HP, Wari V, Willumsen JF (2020). World Health Organization 2020 guidelines on physical activity and sedentary behaviour. Br J Sports Med.

[9] Fihn S, Gardin J, Abrams J (2012). 2012 ACCF/AHA/ACP/AATS/PCNA/SCAI/STS guideline for the diagnosis and management of patients with stable ischemic heart disease: executive summary: a report of the American College of Cardiology Foundation/American Heart Association task force on practice guidelines, and the American College of Physicians, American Association for Thoracic Surgery, Preventive Cardiovascular Nurses Association, Society for Cardiovascular Angiography and Interventions, and Society of Thoracic Surgeons. J Am Coll Cardiol.

[10] Dibben GO, Dalal HM, Taylor RS, Doherty P, Tang LH, Hillsdon M (2018). Cardiac rehabilitation and physical activity: systematic review and meta-analysis. Heart.

[11] Freene N, McManus M, Mair T (2018). Objectively measured changes in physical activity and sedentary behavior in cardiac rehabilitation: a prospective cohort study. J Cardiopulm Rehabil Prev.

[12] Evenson K, Butler E, Rosamond W (2014). Prevalence of physical activity and sedentary behavior among adults with cardiovascular disease in the United States. J Cardiopulm Rehabil Prev.

[13] Vasankari V, Halonen J, Vasankari T, Anttila V, Airaksinen J, Sievänen Harri, Hartikainen J (2021). Physical activity and sedentary behaviour in secondary prevention of coronary artery disease: A review. Am J Prev Cardiol.

[14] Eysenbach G (2001). What is e-health?. J Med Internet Res.

[15] Hutchesson MJ, Gough C, Müller Andre Matthias, Short CE, Whatnall MC, Ahmed M, Pearson N, Yin Z, Ashton LM, Maher C, Staiano AE, Mauch CE, DeSmet A, Vandelanotte C (2021). eHealth interventions targeting nutrition, physical activity, sedentary behavior, or obesity in adults: A scoping review of systematic reviews. Obes Rev.

[16] Widmer RJ, Collins NM, Collins CS, West CP, Lerman LO, Lerman A (2015). Digital health interventions for the prevention of cardiovascular disease: a systematic review and meta-analysis. Mayo Clin Proc.

[17] van Heerden A, Tomlinson M, Swartz L (2012). Point of care in your pocket: a research agenda for the field of m-health. Bull World Health Org.

[18] Cotie LM, Prince SA, Elliott CG, Ziss MC, McDonnell LA, Mullen KA, Hiremath S, Pipe AL, Reid RD, Reed JL (2018). The effectiveness of eHealth interventions on physical activity and measures of obesity among working-age women: a systematic review and meta-analysis. Obes Rev.

[19] Murray E, Khadjesari Z, White IR, Kalaitzaki E, Godfrey C, McCambridge J, Thompson SG, Wallace P (2009). Methodological challenges in online trials. J Med Internet Res.

[20] Ashur C, Cascino T, Lewis C (2021). Do wearable activity trackers increase physical activity among cardiac rehabilitation participants? A systematic review and meta-analysis. J Cardiopulm Rehabil Prev 2021.

[21] Duan Y, Shang B, Liang W, Du G, Yang M, Rhodes RE (2021). Effects of eHealth-Based Multiple Health Behavior Change Interventions on Physical Activity, Healthy Diet, and Weight in People With Noncommunicable Diseases: Systematic Review and Meta-analysis. J Med Internet Res.

[22] Kaminsky L, Brubaker P, Guazzi M (2016). Assessing physical activity as a core component in cardiac rehabilitation: a position statement of the American Association of Cardiovascular and Pulmonary Rehabilitation. J Cardiopulm Rehabil Prev.

[23] Patterson K, Davey R, Keegan R, Freene N (2021). Smartphone applications for physical activity and sedentary behaviour change in people with cardiovascular disease: A systematic review and meta-analysis. PLoS One.

[24] Laukkanen JA, Isiozor NM, Kunutsor SK (2022). Objectively Assessed Cardiorespiratory Fitness and All-Cause Mortality Risk: An Updated Meta-analysis of 37 Cohort Studies Involving 2,258,029 Participants. Mayo Clinic Proceedings.

[25] Vanhees L, Fagard R, Thijs L, Amery A (1995). Prognostic value of training-induced change in peak exercise capacity in patients with myocardial infarcts and patients with coronary bypass surgery. Am J Cardiol.

[26] Carbone S, Kim Y, Kachur S, Billingsley Hayley, Kenyon Jonathan, De Schutter Alban, Milani Richard V, Lavie Carl J (2022). Peak oxygen consumption achieved at the end of cardiac rehabilitation predicts long-term survival in patients with coronary heart disease. Eur Heart J Qual Care Clin Outcomes.

[27] Zhao H, Ma J, Zhou Q, Chen W, Zhu W, Cai Z, Lei H, Deng Y, Xu L, Qiu J (2016). Investigating the differences of body mass index and waist circumference in the follow-up assessment of patients to cardiac rehabilitation with acute coronary syndrome. Australas Phys Eng Sci Med.

[28] Bastien M, Poirier P, Lemieux I, Després Jean-Pierre (2014). Overview of epidemiology and contribution of obesity to cardiovascular disease. Prog Cardiovasc Dis.

[29] Ades PA, Savage PD, Toth MJ, Harvey-Berino J, Schneider DJ, Bunn JY, Audelin MC, Ludlow M (2009). High-calorie-expenditure exercise: a new approach to cardiac rehabilitation for overweight coronary patients. Circulation.

[30] Swift DL, Lavie CJ, Johannsen NM, Arena R, Earnest CP, O'Keefe James H, Milani RV, Blair SN, Church TS (2013). Physical activity, cardiorespiratory fitness, and exercise training in primary and secondary coronary prevention. Circ J.

[31] Higgins J, Thomas J Cochrane Handbook for Systematic Reviews of Interventions Version 6.2.0 [updated 2021].

[32] Page MJ, McKenzie JE, Bossuyt PM, Boutron I, Hoffmann TC, Mulrow CD, Shamseer L, Tetzlaff JM, Akl EA, Brennan SE, Chou R, Glanville J, Grimshaw JM, Hróbjartsson Asbjørn, Lalu MM, Li T, Loder EW, Mayo-Wilson E, McDonald S, McGuinness LA, Stewart LA, Thomas J, Tricco AC, Welch VA, Whiting P, Moher D (2021). The PRISMA 2020 statement: an updated guideline for reporting systematic reviews. BMJ.

[33] Visseren FLJ, Mach F, Smulders YM (2022). 2021 ESC guidelines on cardiovascular disease prevention in clinical practice. Eur J Prev Cardiol.

[34] Higgins JPT, Altman DG, Gøtzsche Peter C, Jüni Peter, Moher D, Oxman AD, Savovic J, Schulz KF, Weeks L, Sterne JAC, Cochrane Bias Methods Group, Cochrane Statistical Methods Group (2011). The Cochrane Collaboration's tool for assessing risk of bias in randomised trials. BMJ.

[35] Fontaine G, Cossette S, Maheu-Cadotte M, Mailhot T, Deschênes Marie-France, Mathieu-Dupuis G (2017). Effectiveness of Adaptive E-Learning Environments on Knowledge, Competence, and Behavior in Health Professionals and Students: Protocol for a Systematic Review and Meta-Analysis. JMIR Res Protoc.

[36] Prince SA, Adamo KB, Hamel ME, Hardt J, Connor Gorber Sarah, Tremblay M (2008). A comparison of direct versus self-report measures for assessing physical activity in adults: a systematic review. Int J Behav Nutr Phys Act.

[37] Richardson M, Garner P, Donegan S (2019). Interpretation of subgroup analyses in systematic reviews: A tutorial. Clinical Epidemiology and Global Health.

[38] Guyatt GH, Oxman AD, Vist GE, Kunz R, Falck-Ytter Y, Alonso-Coello P, Schünemann Holger J, GRADE Working Group (2008). GRADE: an emerging consensus on rating quality of evidence and strength of recommendations. BMJ.

[39] Balshem H, Helfand M, Schünemann Holger J, Oxman AD, Kunz R, Brozek J, Vist GE, Falck-Ytter Y, Meerpohl J, Norris S, Guyatt GH (2011). GRADE guidelines: 3. Rating the quality of evidence. J Clin Epidemiol.

[40] Hakala S, Kivistö Heikki, Paajanen T, Kankainen A, Anttila M, Heinonen A, Sjögren Tuulikki (2021). Effectiveness of Distance Technology in Promoting Physical Activity in Cardiovascular Disease Rehabilitation: Cluster Randomized Controlled Trial, A Pilot Study. JMIR Rehabil Assist Technol.

[41] Reid RD, Wooding EA, Blanchard CM, Moghei M, Harris J, Proulx G, Prince SA, Mullen KA, Ghisi GM, Krahn M, Chessex C, Pipe AL, Mark AE, Grace SL (2021). A Randomized Controlled Trial of an Exercise Maintenance Intervention in Men and Women After Cardiac Rehabilitation (ECO-PCR Trial). Can J Cardiol.

[42] Engelen MM, van Dulmen S, Puijk-Hekman S, Vermeulen H, Nijhuis-van der Sanden MW, Bredie SJ, van Gaal BG (2020). Evaluation of a Web-Based Self-Management Program for Patients With Cardiovascular Disease: Explorative Randomized Controlled Trial. J Med Internet Res.

[43] Claes Jomme, Cornelissen Véronique, McDermott Clare, Moyna Niall, Pattyn Nele, Cornelis Nils, Gallagher Anne, McCormack Ciara, Newton Helen, Gillain Alexandra, Budts Werner, Goetschalckx Kaatje, Woods Catherine, Moran Kieran, Buys Roselien (2020). Feasibility, Acceptability, and Clinical Effectiveness of a Technology-Enabled Cardiac Rehabilitation Platform (Physical Activity Toward Health-I): Randomized Controlled Trial. J Med Internet Res.

[44] Barnason S, Zimmerman L, Schulz P, Pullen C, Schuelke S (2019). Weight management telehealth intervention for overweight and obese rural cardiac rehabilitation participants: A randomised trial. J Clin Nurs.

[45] Maddison R, Rawstorn JC, Stewart RAH, Benatar J, Whittaker R, Rolleston A, Jiang Y, Gao L, Moodie M, Warren I, Meads A, Gant N (2019). Effects and costs of real-time cardiac telerehabilitation: randomised controlled non-inferiority trial. Heart.

[46] Prince SA, Reed JL, Cotie LM, Harris J, Pipe AL, Reid RD (2018). Results of the Sedentary Intervention Trial in Cardiac Rehabilitation (SIT-CR Study): A pilot randomized controlled trial. Int J Cardiol.

[47] Duscha BD, Piner LW, Patel MP, Craig KP, Brady M, McGarrah RW, Chen C, Kraus WE (2018). Effects of a 12-week mHealth program on peak VO2 and physical activity patterns after completing cardiac rehabilitation: A randomized controlled trial. Am Heart J.

[48] Salvi D, Ottaviano M, Muuraiskangas S, Martínez-Romero A, Vera-Muñoz C, Triantafyllidis A, Cabrera Umpiérrez MF, Arredondo Waldmeyer MT, Skobel E, Knackstedt C, Liedes H, Honka A, Luprano J, Cleland JG, Stut W, Deighan C (2017). An m-Health system for education and motivation in cardiac rehabilitation: the experience of HeartCycle guided exercise. J Telemed Telecare.

[49] Young L, Hertzog M, Barnason S (2016). Effects of a home-based activation intervention on self-management adherence and readmission in rural heart failure patients: the PATCH randomized controlled trial. BMC Cardiovasc Disord.

[50] Alsaleh E, Windle R, Blake H (2016). Behavioural intervention to increase physical activity in adults with coronary heart disease in Jordan. BMC Public Health.

[51] Frederix I, Hansen D, Coninx K, Vandervoort P, Vandijck D, Hens N, Van Craenenbroeck E, Van Driessche N, Dendale P (2015). Medium-Term Effectiveness of a Comprehensive Internet-Based and Patient-Specific Telerehabilitation Program With Text Messaging Support for Cardiac Patients: Randomized Controlled Trial. J Med Internet Res.

[52] Guiraud T, Granger R, Gremeaux V, Bousquet M, Richard L, Soukarié Laurent, Babin T, Labrunée Marc, Sanguignol F, Bosquet L, Golay A, Pathak A (2012). Telephone support oriented by accelerometric measurements enhances adherence to physical activity recommendations in noncompliant patients after a cardiac rehabilitation program. Arch Phys Med Rehabil.

[53] Reid RD, Morrin LI, Beaton LJ, Papadakis S, Kocourek J, McDonnell L, Slovinec D'Angelo Monika E, Tulloch H, Suskin N, Unsworth K, Blanchard C, Pipe AL (2012). Randomized trial of an internet-based computer-tailored expert system for physical activity in patients with heart disease. Eur J Prev Cardiol.

[54] Reid RD, Morrin LI, Higginson LA, Wielgosz A, Blanchard C, Beaton LJ, Nelson C, McDonnell L, Oldridge N, Wells GA, Pipe AL (2012). Motivational counselling for physical activity in patients with coronary artery disease not participating in cardiac rehabilitation. Eur J Prev Cardiol.

[55] Barnason S, Zimmerman L, Nieveen J, Schulz P, Miller C, Hertzog M, Tu C (2009). Influence of a symptom management telehealth intervention on older adults' early recovery outcomes after coronary artery bypass surgery. Heart Lung.

[56] Chan NPT, Lai AYK, Choy HK, Cheung DYT, Wan ANT, Cheng VYH, Chan KY, Lau YK, Yung CY, Cheung GOC, Lam TH (2022). Feasibility and Potential Effectiveness of a Smartphone Zero-Time Exercise Intervention for Promoting Physical Activity and Fitness in Patients With Coronary Heart Disease: A Pilot Randomized Controlled Trial. Front Public Health.

[57] Patel MS, Bachireddy C, Small DS, Harrison JD, Harrington TO, Oon AL, Rareshide CAL, Snider CK, Volpp KG (2021). Effect of Goal-Setting Approaches Within a Gamification Intervention to Increase Physical Activity Among Economically Disadvantaged Adults at Elevated Risk for Major Adverse Cardiovascular Events: The ENGAGE Randomized Clinical Trial. JAMA Cardiol.

[58] Kayser JW, Cossette S, Côté José, Tanguay J, Tremblay J, Diodati JG, Bourbonnais A, Purden M, Juneau M, Terrier J, Dupuis J, Maheu-Cadotte Marc-André, Fontaine G, Cournoyer D (2019). A web-based tailored nursing intervention (TAVIE en m@rche) aimed at increasing walking after an acute coronary syndrome: Multicentre randomized trial. J Adv Nurs.

[59] Antypas K, Wangberg SC (2014). An Internet- and mobile-based tailored intervention to enhance maintenance of physical activity after cardiac rehabilitation: short-term results of a randomized controlled trial. J Med Internet Res.

[60] Avila A, Claes J, Goetschalckx K, Buys R, Azzawi M, Vanhees L, Cornelissen V (2018). Home-Based Rehabilitation With Telemonitoring Guidance for Patients With Coronary Artery Disease (Short-Term Results of the TRiCH Study): Randomized Controlled Trial. J Med Internet Res.

[61] Dalli Peydró Ernesto, Sanz Sevilla N, Tuzón Segarra María T, Miró Palau Vicente, Sánchez Torrijos Jorge, Cosín Sales Juan (2022). A randomized controlled clinical trial of cardiac telerehabilitation with a prolonged mobile care monitoring strategy after an acute coronary syndrome. Clin Cardiol.

[62] Devi R, Powell J, Singh S (2014). A web-based program improves physical activity outcomes in a primary care angina population: randomized controlled trial. J Med Internet Res.

[63] Ozemek C, Strath S, Riggin K (2020). Pedometer feedback interventions increase daily physical activity in phase III cardiac rehabilitation participants. J Cardiopulm Rehabil Prev 2020.

[64] Kaminsky L, Jones J, Riggin K (2013). A pedometer-based physical activity intervention for patients entering a maintenance cardiac rehabilitation program: a pilot study. Cardiovasc Diagn Ther.

[65] Pinto R, Pires ML, Borges M, Pinto ML, Sousa Guerreiro C, Miguel S, Santos O, Ricardo I, Cunha N, Alves da Silva P, Correia AL, Fiúza Sílvia, Caldeira E, Salazar F, Rodrigues C, Cordeiro Ferreira M, Afonso G, Araújo Graça, Martins J, Ramalhinho M, Sousa P, Pires S, Jordão Alda, Pinto FJ, Abreu A (2022). Digital home-based multidisciplinary cardiac rehabilitation: How to counteract physical inactivity during the COVID-19 pandemic. Rev Port Cardiol.

[66] Legler S, Celano CM, Beale EE, Hoeppner BB, Huffman JC (2020). Use of text messages to increase positive affect and promote physical activity in patients with heart disease: The Promoting Activity in Cardiac Patients via Text Messages (PACT) pilot study. Curr Psychol.

[67] Freene N, van Berlo S, McManus M, Mair T, Davey R (2020). A Behavioral Change Smartphone App and Program (ToDo-CR) to Decrease Sedentary Behavior in Cardiac Rehabilitation Participants: Prospective Feasibility Cohort Study. JMIR Form Res.

[68] Sengupta A, Beckie T, Dutta K, Dey A, Chellappan S (2020). A Mobile Health Intervention System for Women With Coronary Heart Disease: Usability Study. JMIR Form Res.

[69] Wongvibulsin S, Habeos EE, Huynh PP, Xun H, Shan R, Porosnicu Rodriguez KA, Wang J, Gandapur YK, Osuji N, Shah LM, Spaulding EM, Hung G, Knowles K, Yang WE, Marvel FA, Levin E, Maron DJ, Gordon NF, Martin SS (2021). Digital Health Interventions for Cardiac Rehabilitation: Systematic Literature Review. J Med Internet Res.

[70] Arem H, Moore SC, Patel A, Hartge P, Berrington de Gonzalez A, Visvanathan K, Campbell PT, Freedman M, Weiderpass E, Adami HO, Linet MS, Lee I, Matthews CE (2015). Leisure time physical activity and mortality: a detailed pooled analysis of the dose-response relationship. JAMA Intern Med.

[71] Su JJ, Yu DSF, Paguio JT (2020). Effect of eHealth cardiac rehabilitation on health outcomes of coronary heart disease patients: A systematic review and meta-analysis. J Adv Nurs.

[72] Knaeps S, Bourgois JG, Charlier R, Mertens E, Lefevre J, Wijndaele K (2018). Ten-year change in sedentary behaviour, moderate-to-vigorous physical activity, cardiorespiratory fitness and cardiometabolic risk: independent associations and mediation analysis. Br J Sports Med.

[73] Mikkelsen N, Cadarso-Suárez Carmen, Lado-Baleato O, Díaz-Louzao Carla, Gil CP, Reeh J, Rasmusen H, Prescott E (2020). Improvement in VO2peak predicts readmissions for cardiovascular disease and mortality in patients undergoing cardiac rehabilitation. Eur J Prev Cardiol.

[74] Flint AJ, Rexrode KM, Hu FB, Glynn RJ, Caspard H, Manson JE, Willett WC, Rimm EB (2010). Body mass index, waist circumference, and risk of coronary heart disease: a prospective study among men and women. Obes Res Clin Pract.

[75] Ades P, Savage P, Harvey-Berino J (2010). The treatment of obesity in cardiac rehabilitation. J Cardiopulm Rehabil Prev.

[76] Rawstorn JC, Gant N, Direito A, Beckmann C, Maddison R (2016). Telehealth exercise-based cardiac rehabilitation: a systematic review and meta-analysis. Heart.

[77] Peçanha Tiago, Goessler KF, Roschel H, Gualano B (2020). Social isolation during the COVID-19 pandemic can increase physical inactivity and the global burden of cardiovascular disease. Am J Physiol Heart Circ Physiol.

[78] Scherrenberg M, Wilhelm M, Hansen D, Völler Heinz, Cornelissen V, Frederix I, Kemps H, Dendale P (2020). The future is now: a call for action for cardiac telerehabilitation in the COVID-19 pandemic from the secondary prevention and rehabilitation section of the European Association of Preventive Cardiology. Eur J Prev Cardiol.

[79] Kebapci A, Ozkaynak M, Lareau SC (2020). Effects of eHealth-Based Interventions on Adherence to Components of Cardiac Rehabilitation: A Systematic Review. J Cardiovasc Nurs.

[80] Jin K, Khonsari S, Gallagher R, Gallagher P, Clark AM, Freedman B, Briffa T, Bauman A, Redfern J, Neubeck L (2019). Telehealth interventions for the secondary prevention of coronary heart disease: A systematic review and meta-analysis. Eur J Cardiovasc Nurs.

[81] Hupin D, Roche F, Gremeaux V, Chatard J, Oriol M, Gaspoz J, Barthélémy Jean-Claude, Edouard P (2015). Even a low-dose of moderate-to-vigorous physical activity reduces mortality by 22% in adults aged ≥60 years: a systematic review and meta-analysis. Br J Sports Med.

