# Supplementary material for: Effectiveness of eHealth Interventions on Moderate-to-Vigorous Intensity Physical Activity Among Patients in Cardiac Rehabilitation: Systematic Review and Meta-analysis
Source: J Med Internet Res. 2023 Mar 29;25:e42845. doi: 10.2196/42845 (PMC10131595; doi:10.2196/42845)
Supplement: Multimedia Appendix 1 [file jmir_v25i1e42845_app1.docx]

**Multimedia Appendix 1**

Amendments to information provided at registration.

With the language restrictions removed, we are updating the search on 27 November 2022. We extended the type of included studies from randomized controlled trials to experimental studies. For secondary outcome measures, we only focused on three of them: cardiorespiratory fitness, waist circumference, and systolic blood pressure. We changed the software for the statistical analysis from Review Manager 5.4.1 to Stata BE, version 17. We also examined differences between subgroups in sample size, intervention characteristics, intervention duration, interaction with health care professionals, and control characteristics for prior determined subgroup analyses. Based on the intervention components of the included studies, we modified the subgroup analysis for different intervention delivery methods into three subgroups, wearable-based, web-based, and communication-based.
