# Supplementary material for: Effectiveness of eHealth Interventions on Moderate-to-Vigorous Intensity Physical Activity Among Patients in Cardiac Rehabilitation: Systematic Review and Meta-analysis
Source: J Med Internet Res. 2023 Mar 29;25:e42845. doi: 10.2196/42845 (PMC10131595; doi:10.2196/42845)
Supplement: Multimedia Appendix 3 [file jmir_v25i1e42845_app3.docx]

**Multimedia Appendix 3**

Search strategy.

This Supplementary file provides the search strategy details, performed November 27, 2022.

| PubMed | |
| --- | --- |
| #1 | eHealth[Title/Abstract] OR e-Health[Title/Abstract] |
| #2 | Cell Phone[MeSH Terms] |
| #3 | Text Messaging[MeSH Terms] |
| #4 | Internet[MeSH Terms] |
| #5 | ((cell*[Title/Abstract] AND phone*[Title/Abstract]) OR (mobile[Title/Abstract] AND phone*[Title/Abstract])) OR (smart[Title/Abstract] AND phone*[Title/Abstract]) |
| #6 | text messag*[Title/Abstract] |
| #7 | smartphone*[Title/Abstract] OR iPhone*[Title/Abstract] |
| #8 | (((((((((((mobile[Title/Abstract] AND app[Title/Abstract]) OR (mobile[Title/Abstract] AND apps[Title/Abstract])) OR (mobile[Title/Abstract] AND application*[Title/Abstract])) OR (phone[Title/Abstract] AND app[Title/Abstract])) OR (phone[Title/Abstract] AND apps[Title/Abstract])) OR (phone[Title/Abstract] AND application*[Title/Abstract])) OR (iPhone[Title/Abstract] AND app[Title/Abstract])) OR (iPhone[Title/Abstract] AND apps[Title/Abstract])) OR (iPhone[Title/Abstract] AND application*[Title/Abstract])) OR (smartphone[Title/Abstract] AND app[Title/Abstract])) OR (smartphone[Title/Abstract] AND apps[Title/Abstract])) OR (smartphone[Title/Abstract] AND application*[Title/Abstract]) |
| #9 | internet[Title/Abstract] |
| #10 | web-based[Title/Abstract] |
| #11 | Computer-Assisted Instruction[MeSH Terms] |
| #12 | computer-tailored[Title/Abstract] |
| #13 | (((mobile[Title/Abstract] AND health[Title/Abstract]) OR (mobile[Title/Abstract] AND tech*[Title/Abstract])) OR (mobile[Title/Abstract] AND device*[Title/Abstract])) OR (mobile[Title/Abstract] AND telephone*[Title/Abstract]) |
| #14 | exergam*[Title/Abstract] OR exer-gam*[Title/Abstract] |
| #15 | Wii[Title/Abstract] |
| #16 | (((((((((((fitness[Title/Abstract] AND videogam*[Title/Abstract]) OR (fitness[Title/Abstract] AND "video gam*"[Title/Abstract])) OR (fitness[Title/Abstract] AND gaming[Title/Abstract])) OR (activ*[Title/Abstract] AND videogam*[Title/Abstract])) OR (activ*[Title/Abstract] AND "video gam*"[Title/Abstract])) OR (activ*[Title/Abstract] AND gaming[Title/Abstract])) OR (exercis*[Title/Abstract] AND videogam*[Title/Abstract])) OR (exercis*[Title/Abstract] AND "video gam*"[Title/Abstract])) OR (exercis*[Title/Abstract] AND gaming[Title/Abstract])) OR (interactive[Title/Abstract] AND gaming[Title/Abstract])) OR (interactive[Title/Abstract] AND "video gam*"[Title/Abstract])) OR (interactive[Title/Abstract] AND videogam*[Title/Abstract]) |
| #17 | Accelerometry[MeSH Terms] |
| #18 | acceleromet*[Title/Abstract] |
| #19 | pedomet*[Title/Abstract] |
| #20 | Electronic Mail[MeSH Terms] |
| #21 | email*[Title/Abstract] OR e-mail*[Title/Abstract] OR "electronic mail*"[Title/Abstract] |
| #22 | (((((((((wearable*[Title/Abstract] AND monitor*[Title/Abstract]) OR (wearable*[Title/Abstract] AND sensor*[Title/Abstract])) OR (wearable*[Title/Abstract] AND sensing[Title/Abstract])) OR (wearable*[Title/Abstract] AND device*[Title/Abstract])) OR (wearable*[Title/Abstract] AND tech*[Title/Abstract])) OR (wear-able*[Title/Abstract] AND monitor*[Title/Abstract])) OR (wear-able*[Title/Abstract] AND sensor*[Title/Abstract])) OR (wear-able*[Title/Abstract] AND sensing[Title/Abstract])) OR (wear-able*[Title/Abstract] AND device*[Title/Abstract])) OR (wear-able*[Title/Abstract] AND tech*[Title/Abstract]) |
| #23 | ((activity[Title/Abstract] AND monitor[Title/Abstract]) OR (activity[Title/Abstract] AND monitors[Title/Abstract])) OR (activity[Title/Abstract] AND sensor*[Title/Abstract]) |
| #24 | ((motion[Title/Abstract] AND sensor*[Title/Abstract]) OR (motion[Title/Abstract] AND monitor[Title/Abstract])) OR (motion[Title/Abstract] AND monitors[Title/Abstract]) |
| #25 | fitbit[Title/Abstract] |
| #26 | activPal*[Title/Abstract] |
| #27 | MyFitnessPal[Title/Abstract] |
| #28 | NikeFuel[Title/Abstract] |
| #29 | Sensewear[Title/Abstract] |
| #30 | Omron[Title/Abstract] |
| #31 | m-health[Title/Abstract] OR mHealth[Title/Abstract] |
| #32 | personal digital assistant*[Title/Abstract] |
| #33 | pda[Title/Abstract] |
| #34 | Computers, Handheld[MeSH Terms] |
| #35 | Telemedicine[MeSH Terms] |
| #36 | #1 OR #2 OR #3 OR #4 OR #5 OR #6 OR #7 OR #8 OR #9 OR #10 OR #11 OR #12 OR #13 OR #14 OR #15 OR #16 OR #17 OR #18 OR #19 OR #20 OR #21 OR #22 OR #23 OR #24 OR #25 OR #26 OR #27 OR #28 OR #29 OR #30 OR #31 OR #32 OR #33 OR #34 OR #35 |
| #37 | Motor Activity[MeSH Terms] |
| #38 | (((((((((((((((((Exercise[MeSH Terms]) OR (Cool-Down Exercise[MeSH Terms])) OR (Gymnastics[MeSH Terms])) OR (Muscle Stretching Exercises[MeSH Terms])) OR (Physical Conditioning, Human[MeSH Terms])) OR (Circuit-Based Exercise[MeSH Terms])) OR (Endurance Training[MeSH Terms])) OR (High-Intensity Interval Training[MeSH Terms])) OR (Plyometric Exercise[MeSH Terms])) OR (Resistance Training[MeSH Terms])) OR (Preoperative Exercise[MeSH Terms])) OR (Running[MeSH Terms])) OR (Jogging[MeSH Terms])) OR (Marathon Running[MeSH Terms])) OR (Swimming[MeSH Terms])) OR (Walking[MeSH Terms])) OR (Stair Climbing[MeSH Terms])) OR (Warm-Up Exercise[MeSH Terms]) |
| #39 | Physical Fitness[MeSH Terms] |
| #40 | "Physical Education and Training"[MeSH Terms] |
| #41 | (((((Exercise Therapy[MeSH Terms]) OR (Endurance Training[MeSH Terms])) OR (Motion Therapy, Continuous Passive[MeSH Terms])) OR (Muscle Stretching Exercises[MeSH Terms])) OR (Plyometric Exercise[MeSH Terms])) OR (Resistance Training[MeSH Terms]) |
| #42 | Movement[MeSH Terms] |
| #43 | Bicycling[MeSH Terms] |
| #44 | ((physical*[Title/Abstract] AND activit*[Title/Abstract]) OR (physical*[Title/Abstract] AND exercise*[Title/Abstract])) OR (physical*[Title/Abstract] AND fitness[Title/Abstract]) |
| #45 | (((((((fitness[Title/Abstract] AND class*[Title/Abstract]) OR (fitness[Title/Abstract] AND course*[Title/Abstract])) OR (fitness[Title/Abstract] AND program*[Title/Abstract])) OR (fitness[Title/Abstract] AND training[Title/Abstract])) OR (exercise[Title/Abstract] AND class*[Title/Abstract])) OR (exercise[Title/Abstract] AND course*[Title/Abstract])) OR (exercise[Title/Abstract] AND program*[Title/Abstract])) OR (exercise[Title/Abstract] AND training[Title/Abstract]) |
| #46 | ("aerobic exercis*"[Title/Abstract]) OR (aerobics[Title/Abstract]) |
| #47 | ((((((walk*[Title/Abstract]) OR (run[Title/Abstract])) OR (bike[Title/Abstract])) OR (bicycl*[Title/Abstract])) OR (runs[Title/Abstract])) OR (running[Title/Abstract])) OR (runnings[Title/Abstract]) |
| #48 | yoga[Title/Abstract] |
| #49 | Yoga[MeSH Terms] |
| #50 | ((moderate[Title/Abstract] AND "intensity activit*"[Title/Abstract]) OR (high[Title/Abstract] AND "intensity activit*"[Title/Abstract])) OR (vigorous[Title/Abstract] AND "intensity activit*"[Title/Abstract]) |
| #51 | moderate-vigorous[Title/Abstract] AND activit*[Title/Abstract] |
| #52 | "moderate to vigorous"[Title/Abstract] AND activit*[Title/Abstract] |
| #53 | mvpa[Title/Abstract] |
| #54 | exercise*[Title/Abstract] |
| #55 | #37 OR #38 OR #39 OR #40 OR #41 OR #42 OR #43 OR #44 OR #45 OR #46 OR #47 OR #48 OR #49 OR #50 OR #51 OR #52 OR #53 OR #54 |
| #56 | intervention*[Title/Abstract] |
| #57 | Program Evaluation[MeSH Terms] |
| #58 | Evaluation Studies as Topic[MeSH Terms] OR Evaluation Study [Publication Type] |
| #59 | Multicenter Studies as Topic[MeSH Terms] OR Multicenter Study [Publication Type] |
| #60 | Observational Studies as Topic[MeSH Terms] OR Observational Study [Publication Type] |
| #61 | ((observational[Title/Abstract]) AND (study[Title/Abstract])) OR ((observational[Title/Abstract]) AND (studies[Title/Abstract])) |
| #62 | Randomized Controlled Trials as Topic[MeSH Terms] |
| #63 | Randomized Controlled Trial[Publication Type] |
| #64 | random*[Title/Abstract] |
| #65 | Clinical Trials as Topic[MeSH Terms] OR Clinical Trial [Publication Type] |
| #66 | Controlled Clinical Trials as Topic[MeSH Terms] OR Controlled Clinical Trial [Publication Type] |
| #67 | (clinical[Title/Abstract]) AND (trial*[Title/Abstract]) |
| #68 | Case-Control Studies[MeSH Terms] |
| #69 | Cohort studies[MeSH Terms] |
| #70 | #56 OR #57 OR #58 OR #59 OR #60 OR #61 OR #62 OR #63 OR #64 OR #65 OR #66 OR #67 OR #68 OR #69 |
| #71 | Myocardial Infarction[MeSH Terms] |
| #72 | Myocardial Ischemia[MeSH Terms] |
| #73 | Coronary Disease[MeSH Terms] |
| #74 | Coronary Artery Bypass[MeSH Terms] |
| #75 | Heart Bypass, Left[MeSH Terms] |
| #76 | Heart Bypass, Right[MeSH Terms] |
| #77 | Myocardial Revascularization[MeSH Terms] |
| #78 | Heart Failure[MeSH Terms] |
| #79 | Heart Diseases[MeSH Terms] |
| #80 | coronary[Title/Abstract] |
| #81 | myocard*[Title/Abstract] |
| #82 | cardiac*[Title/Abstract] |
| #83 | heart attack*[Title/Abstract] |
| #84 | heart infarct*[Title/Abstract] |
| #85 | angina[Title/Abstract] |
| #86 | heart failure[Title/Abstract] |
| #87 | heart disease*[Title/Abstract] |
| #88 | CABG[Title/Abstract] |
| #89 | PTCA[Title/Abstract] |
| #90 | ((heart[Title/Abstract]) OR (cardiac[Title/Abstract])) AND (stent*[Title/Abstract]) |
| #91 | heart bypass[Title/Abstract] |
| #92 | postmyocard*[Title/Abstract] |
| #93 | #71 OR #72 OR #73 OR #74 OR #75 OR #76 OR #77 OR #78 OR #79 OR #80 OR #81 OR #82 OR #83 OR #84 OR #85 OR #86 OR #87 OR #88 OR #89 OR #90 OR #91 OR #92 |
| #94 | rehab*[Title/Abstract] |
| #95 | Rehabilitation[MeSH Terms] |
| #96 | #94 OR #95 |
| #97 | #93 AND #96 |
| #98 | #36 AND #55 AND #70 AND #97 |
| #99 | Filters applied: Humans. |

| Web of Science | |
| --- | --- |
| #1 | TS=(eHealth OR e-Health) |
| #2 | TS=(Cell Phone) |
| #3 | TS=(Text Messaging) |
| #4 | TS=(Internet) |
| #5 | ((TS=(cell* NEAR/1 phone*)) OR TS=(mobile NEAR/1 phone*)) OR TS=(smart NEAR/1 phone*) |
| #6 | TS=(text messag*) |
| #7 | TS=(smartphone* OR iPhone*) |
| #8 | TS=((mobile or phone or iPhone or smartphone) NEAR/2 (app or apps or application*)) |
| #9 | TS=(internet) |
| #10 | TS=(web-based) |
| #11 | TS=(Computer-Assisted Instruction) |
| #12 | TS=(computer-tailored) |
| #13 | TS=(mobile NEAR/1 (health or tech* or device* or telephone*)) |
| #14 | TS=(exergam* OR exer-gam*) |
| #15 | TS=(Wii) |
| #16 | TS=((fitness or activ* or exercis* or interactive) NEAR/2 (videogam* or "video gam*" or gaming)) |
| #17 | TS=(Accelerometry) |
| #18 | TS=(acceleromet*) |
| #19 | TS=(pedomet*) |
| #20 | TS=(Electronic Mail) |
| #21 | TS=(email* or e-mail* or "electronic mail*") |
| #22 | TS=((wearable* or wear-able*) NEAR/3 (monitor* or sensor* or sensing or device* or tech*)) |
| #23 | TS=(activity NEAR/1 (monitor or monitors or sensor*)) |
| #24 | TS=(motion NEAR/1 (sensor* or monitor or monitors)) |
| #25 | TS=(fitbit) |
| #26 | TS=(activPal*) |
| #27 | TS=(MyFitnessPal) |
| #28 | TS=(NikeFuel) |
| #29 | TS=(Sensewear) |
| #30 | TS=(Omron) |
| #31 | TS=(mHealth or m-Health) |
| #32 | TS=(personal digital assistant*) |
| #33 | TS=(pda) |
| #34 | TS=(Computers, Handheld) |
| #35 | TS=(telemedicine) |
| #36 | #1 OR #2 OR #3 OR #4 OR #5 OR #6 OR #7 OR #8 OR #9 OR #10 OR #11 OR #12 OR #13 OR #14 OR #15 OR #16 OR #17 OR #18 OR #19 OR #20 OR #21 OR #22 OR #23 OR #24 OR #25 OR #26 OR #27 OR #28 OR #29 OR #30 OR #31 OR #32 OR #33 OR #34 OR #35 |
| #37 | TS=(Motor Activity) |
| #38 | (((((((((((((((((TS=(Exercise)) OR TS=(Cool-Down Exercise)) OR TS=(Gymnastics)) OR TS=(Muscle Stretching Exercises)) OR TS=(Physical Conditioning, Human)) OR TS=(Circuit-Based Exercise)) OR TS=(Endurance Training)) OR TS=(High-Intensity Interval Training)) OR TS=(Plyometric Exercise)) OR TS=(Resistance Training)) OR TS=(Preoperative Exercise)) OR TS=(Running)) OR TS=(Jogging)) OR TS=(Marathon Running)) OR TS=(Swimming)) OR TS=(Walking)) OR TS=(Stair Climbing)) OR TS=(Warm-Up Exercise) |
| #39 | TS=(Physical Fitness) |
| #40 | TS=("Physical Education and Training") |
| #41 | (((((TS=(Exercise Therapy)) OR TS=(Endurance Training)) OR TS=(Motion Therapy, Continuous Passive)) OR TS=(Muscle Stretching Exercises)) OR TS=(Plyometric Exercise)) OR TS=(Resistance Training) |
| #42 | TS=(Movement) |
| #43 | TS=(Bicycling) |
| #44 | TS=(physical* NEAR/1 (activit* or exercise* or fitness)) |
| #45 | TS=((fitness or exercise) NEAR/1 (class* or course* or program* or training)) |
| #46 | TS=("aerobic exercis*" or aerobics) |
| #47 | TS=(walk* or run* or bike or bicycl*) |
| #48 | TS=(yoga) |
| #49 | TS=((moderate or high or vigorous) NEAR/1 "intensity activit*") |
| #50 | TS=(moderate-vigorous NEAR/2 activit*) |
| #51 | TS=("moderate to vigorous" NEAR/2 activit*) |
| #52 | TS=(mvpa) |
| #53 | #37 OR #38 OR #39 OR #40 OR #41 OR #42 OR #43 OR #44 OR #45 OR #46 OR #47 OR #48 OR #49 OR #50 OR #51 OR #52 |
| #54 | TS=(intervention*) |
| #55 | TS=(Program Evaluation) |
| #56 | (TS=(Evaluation Studies as Topic)) OR TS=(Evaluation Study) |
| #57 | (TS=(Multicenter Studies as Topic)) OR TS=(Multicenter Study) |
| #58 | (TS=(Observational Studies as Topic)) OR TS=(Observational Study) |
| #59 | TS=(observational NEAR/1 (study or studies)) |
| #60 | TS=(Randomized Controlled Trials as Topic) |
| #61 | TS=(Randomized Controlled Trial) |
| #62 | TS=(random*) |
| #63 | (TS=(Clinical Trials as Topic)) OR TS=(Clinical Trial) |
| #64 | (TS=(Controlled Clinical Trials as Topic)) OR TS=(Controlled Clinical Trial) |
| #65 | TS=(clinical NEAR/1 trial*) |
| #66 | TS=(Case-Control Studies) |
| #67 | TS=(Cohort studies) |
| #68 | #54 OR #55 OR #56 OR #57 OR #58 OR #59 OR #60 OR #61 OR #62 OR #63 OR #64 OR #65 OR #66 OR #67 |
| #69 | TS=(Myocardial Infarction) |
| #70 | TS=(Myocardial Ischemia) |
| #71 | TS=(Coronary Disease) |
| #72 | TS=(Coronary Artery Bypass) |
| #73 | TS=(Heart Bypass, Left) |
| #74 | TS=(Heart Bypass, Right) |
| #75 | TS=(Myocardial Revascularization) |
| #76 | TS=(Heart Failure) |
| #77 | TS=(Heart Disease*) |
| #78 | TS=(coronary) |
| #79 | TS=(myocard*) |
| #80 | TS=(cardiac*) |
| #81 | TS=(heart attack*) |
| #82 | TS=(heart infarct*) |
| #83 | TS=(angina) |
| #84 | TS=(CABG) |
| #85 | TS=(PTCA) |
| #86 | TS=((heart or cardiac) AND stent*) |
| #87 | TS=(heart bypass) |
| #88 | TS=(postmyocard*) |
| #89 | #69 OR #70 OR #71 OR #72 OR #73 OR #74 OR #75 OR #76 OR #77 OR #78 OR #79 OR #80 OR #81 OR #82 OR #83 OR #84 OR #85 OR #86 OR #87 OR #88 |
| #90 | TS=(rehab*) |
| #91 | TS=(Rehabilitation) |
| #92 | #90 OR #91 |
| #93 | #89 AND #92 |
| #94 | #36 AND #53 AND #68 AND #89 |

| Embase | |
| --- | --- |
| #1 | ehealth:ti,ab,kw OR 'e health':ti,ab,kw |
| #2 | 'cellular phone':ti,ab,kw |
| #3 | 'text messaging'/exp |
| #4 | 'Internet'/exp |
| #5 | ((cell* OR mobile OR smart) NEAR/1 phone*):ti,ab,kw |
| #6 | 'text messag*':ti,ab,kw |
| #7 | smartphone*:ti,ab,kw OR iphone*:ti,ab,kw |
| #8 | ((mobile OR phone OR iphone OR smartphone) NEAR/2 (app OR apps OR application*)):ti,ab,kw |
| #9 | internet:ti,ab,kw |
| #10 | 'web based':ti,ab,kw |
| #11 | 'computer-assisted instruction':ti,ab,kw |
| #12 | 'computer tailored':ti,ab,kw |
| #13 | (mobile NEAR/1 (health OR tech* OR device* OR telephone*)):ti,ab,kw |
| #14 | exergam*:ti,ab,kw OR 'exer gam*':ti,ab,kw |
| #15 | wii:ti,ab,kw |
| #16 | ((fitness OR activ* OR exercis* OR interactive) NEAR/2 (videogam* OR 'video gam*' OR gaming)):ti,ab,kw |
| #17 | 'accelerometry'/exp |
| #18 | acceleromet*:ti,ab,kw |
| #19 | pedomet*:ti,ab,kw |
| #20 | 'electronic mail':ti,ab,kw |
| #21 | email*:ti,ab,kw OR 'e mail*':ti,ab,kw OR 'electronic mail*':ti,ab,kw |
| #22 | ((wearable* OR 'wear able*') NEAR/3 (monitor* OR sensor* OR sensing OR device* OR tech*)):ti,ab,kw |
| #23 | (activity NEAR/1 (monitor OR monitors OR sensor*)):ti,ab,kw |
| #24 | (motion NEAR/1 (sensor* OR monitor OR monitors)):ti,ab,kw |
| #25 | fitbit:ti,ab,kw |
| #26 | activpal*:ti,ab,kw |
| #27 | myfitnesspal:ti,ab,kw |
| #28 | nikefuel:ti,ab,kw |
| #29 | sensewear:ti,ab,kw |
| #30 | omron:ti,ab,kw |
| #31 | mhealth:ti,ab,kw OR 'm health':ti,ab,kw |
| #32 | 'personal digital assistant*':ti,ab,kw |
| #33 | pda:ti,ab,kw |
| #34 | 'computers, handheld':ti,ab,kw |
| #35 | 'telemedicine'/exp |
| #36 | #1 OR #2 OR #3 OR #4 OR #5 OR #6 OR #7 OR #8 OR #9 OR #10 OR #11 OR #12 OR #13 OR #14 OR #15 OR #16 OR #17 OR #18 OR #19 OR #20 OR #21 OR #22 OR #23 OR #24 OR #25 OR #26 OR #27 OR #28 OR #29 OR #30 OR #31 OR #32 OR #33 OR #34 OR #35 |
| #37 | 'motor activity'/exp |
| #38 | 'exercise'/exp OR 'cool-down exercise':ti,ab,kw OR 'gymnastics'/exp OR 'muscle stretching exercises':ti,ab,kw OR 'physical conditioning, human':ti,ab,kw OR 'circuit-based exercise':ti,ab,kw OR 'endurance training'/exp OR 'high-intensity interval training':ti,ab,kw OR 'plyometric exercise':ti,ab,kw OR 'resistance training'/exp OR 'preoperative exercise'/exp OR 'running'/exp OR 'jogging'/exp OR 'marathon running'/exp OR 'swimming'/exp OR 'walking'/exp OR 'stair climbing'/exp OR 'warm-up exercise':ti,ab,kw |
| #39 | 'physical fitness':ti,ab,kw |
| #40 | 'physical education':ti,ab,kw AND training:ti,ab,kw |
| #41 | 'exercise therapy':ti,ab,kw OR 'motion therapy, continuous passive':ti,ab,kw OR 'muscle stretching exercises':ti,ab,kw OR 'plyometric exercise':ti,ab,kw |
| #42 | movement:ti,ab,kw |
| #43 | bicycling:ti,ab,kw |
| #44 | (physical* NEAR/1 (activit* OR exercise* OR fitness)):ti,ab,kw |
| #45 | ((fitness OR exercise) NEAR/1 (class* OR course* OR program* OR training)):ti,ab,kw |
| #46 | 'aerobic exercis*':ti,ab,kw OR aerobics:ti,ab,kw |
| #47 | walk*:ti,ab,kw OR run*:ti,ab,kw OR bike:ti,ab,kw OR bicycl*:ti,ab,kw |
| #48 | yoga:ti,ab,kw |
| #49 | 'yoga'/exp |
| #50 | ((moderate OR high OR vigorous) NEAR/1 'intensity activit*'):ti,ab,kw |
| #51 | ('moderate vigorous' NEAR/2 activit*):ti,ab,kw |
| #52 | ('moderate to vigorous' NEAR/2 activit*):ti,ab,kw |
| #53 | mvpa:ti,ab,kw |
| #54 | exercise:ti,ab,kw |
| #55 | #37 OR #38 OR #39 OR #40 OR #41 OR #42 OR #43 OR #44 OR #45 OR #46 OR #47 OR #48 OR #49 OR #50 OR #51 OR #52 OR #53 OR #54 |
| #56 | intervention*:ti,ab,kw |
| #57 | 'program evaluation'/exp |
| #58 | 'evaluation study'/exp |
| #59 | 'multicenter study'/exp |
| #60 | 'observational study'/exp |
| #61 | (observational NEAR/1 (study OR studies)):ti,ab,kw |
| #62 | 'randomized controlled trial'/exp |
| #63 | 'randomized controlled trial (topic)'/exp |
| #64 | random*:ti,ab,kw |
| #65 | 'clinical trial (topic)'/exp |
| #66 | 'controlled clinical trial'/exp |
| #67 | (clinical NEAR/1 trial*):ti,ab,kw |
| #68 | 'case control study'/exp |
| #69 | 'cohort analysis'/exp |
| #70 | #56 OR #57 OR #58 OR #59 OR #60 OR #61 OR #62 OR #63 OR #64 OR #65 OR #66 OR #67 OR #68 OR #69 |
| #71 | 'heart infarction'/exp |
| #72 | 'heart muscle ischemia'/exp |
| #73 | 'coronary artery bypass surgery'/exp |
| #74 | 'coronary artery disease'/exp |
| #75 | 'heart muscle revascularization'/exp |
| #76 | 'angina pectoris'/exp |
| #77 | 'heart failure'/exp |
| #78 | 'heart disease'/exp |
| #79 | 'extracorporeal circulation'/exp |
| #80 | 'heart attack*':ti,ab,kw OR coronary:ti,ab,kw OR myocard*:ti,ab,kw OR cardiac*:ti,ab,kw OR 'heart infarct*':ti,ab,kw OR angina:ti,ab,kw OR 'heart failure*':ti,ab,kw OR 'heart disease*':ti,ab,kw OR cabg:ti,ab,kw OR ptca:ti,ab,kw OR ((heart:ti,ab,kw OR cardiac:ti,ab,kw) AND stent*:ti,ab,kw) OR 'heart bypass*':ti,ab,kw OR postmyocard*:ti,ab,kw |
| #81 | #71 OR #72 OR #73 OR #74 OR #75 OR #76 OR #77 OR #78 OR #79 OR #80 |
| #82 | rehab*:ti,ab,kw |
| #83 | 'rehabilitation'/exp |
| #84 | #82 OR #83 |
| #85 | #81 AND #84 |
| #86 | #36 AND #55 AND #70 AND #85 |
| #87 | #86 AND 'human'/de |

| The Cochrane Library | |
| --- | --- |
| #1 | (eHealth or e-Health):ti,ab,kw |
| #2 | MeSH descriptor: [Cell Phone] this term only |
| #3 | MeSH descriptor: [Text Messaging] this term only |
| #4 | MeSH descriptor: [Internet] this term only |
| #5 | ((cell* or mobile or smart) NEAR/1 phone*):ti,ab,kw OR (text messag*):ti,ab,kw OR (smartphone* or iPhone*):ti,ab,kw OR ((mobile or phone or iPhone or smartphone) NEAR/2 (app or apps or application*)):ti,ab,kw OR (internet):ti,ab,kw |
| #6 | (web-based):ti,ab,kw |
| #7 | MeSH descriptor: [Computer-Assisted Instruction] this term only |
| #8 | (computer-tailored):ti,ab,kw OR (mobile NEAR/1 (health or tech* or device* or telephone*)):ti,ab,kw OR (exergam* or exer-gam*):ti,ab,kw OR (Wii):ti,ab,kw OR ((fitness or activ* or exercis* or interactive) NEAR/2 (videogam* or "video gam*" or gaming)):ti,ab,kw |
| #9 | MeSH descriptor: [Accelerometry] this term only |
| #10 | (acceleromet*):ti,ab,kw OR (pedomet*):ti,ab,kw |
| #11 | MeSH descriptor: [Electronic Mail] this term only |
| #12 | (email* or e-mail* or "electronic mail*"):ti,ab,kw OR ((wearable* or wear-able*) NEAR/3 (monitor* or sensor* or sensing or device* or tech*)):ti,ab,kw OR (activity NEAR/1 (monitor or monitors or sensor*)):ti,ab,kw OR (motion NEAR/1 (sensor* or monitor or monitors)):ti,ab,kw OR (fitbit):ti,ab,kw |
| #13 | (activPal*):ti,ab,kw OR (MyFitnessPal):ti,ab,kw OR (NikeFuel):ti,ab,kw OR (Sensewear):ti,ab,kw OR (Omron):ti,ab,kw |
| #14 | (mHealth or m-Health):ti,ab,kw OR (personal digital assistant*):ti,ab,kw OR (pda):ti,ab,kw |
| #15 | MeSH descriptor: [Computers, Handheld] this term only |
| #16 | MeSH descriptor: [Telemedicine] this term only |
| #17 | {OR #1-#16} |
| #18 | MeSH descriptor: [Motor Activity] this term only |
| #19 | MeSH descriptor: [Exercise] explode all trees |
| #20 | MeSH descriptor: [Physical Fitness] this term only |
| #21 | ("Physical Education and Training"):ti,ab,kw |
| #22 | MeSH descriptor: [Exercise Therapy] explode all trees |
| #23 | MeSH descriptor: [Movement] this term only |
| #24 | MeSH descriptor: [Bicycling] this term only |
| #25 | (physical* NEAR/1 (activit* or exercise* or fitness)):ti,ab,kw OR ((fitness or exercise) NEAR/1 (class* or course* or program* or training)):ti,ab,kw OR ("aerobic exercis*" or aerobics):ti,ab,kw OR (walk* or run* or bike or bicycl*):ti,ab,kw OR (Yoga):ti,ab,kw |
| #26 | MeSH descriptor: [Yoga] this term only |
| #27 | ((moderate or high or vigorous) NEAR/1 "intensity activit*"):ti,ab,kw OR (moderate-vigorous NEAR/2 activit*):ti,ab,kw OR ("moderate to vigorous" NEAR/2 activit*):ti,ab,kw OR (mvpa):ti,ab,kw OR (exercise):ti,ab,kw |
| #28 | {OR #18-#27} |
| #29 | (intervention*):ti,ab,kw |
| #30 | MeSH descriptor: [Program Evaluation] this term only |
| #31 | MeSH descriptor: [Evaluation Study] this term only |
| #32 | MeSH descriptor: [Multicenter Study] this term only |
| #33 | MeSH descriptor: [Observational Study] this term only |
| #34 | MeSH descriptor: [Observational Studies as Topic] this term only |
| #35 | (observational NEAR/1 (study or studies)):ti,ab,kw |
| #36 | MeSH descriptor: [Randomized Controlled Trials as Topic] this term only |
| #37 | MeSH descriptor: [Randomized Controlled Trial] this term only |
| #38 | (random*):ti,ab,kw |
| #39 | MeSH descriptor: [Clinical Trials as Topic] explode all trees |
| #40 | MeSH descriptor: [Clinical Trial] this term only |
| #41 | MeSH descriptor: [Controlled Clinical Trial] this term only |
| #42 | (clinical NEAR/1 trial*):ti,ab,kw |
| #43 | MeSH descriptor: [Case-Control Studies] this term only |
| #44 | MeSH descriptor: [Cohort Studies] explode all trees |
| #45 | {OR #29-#44} |
| #46 | MeSH descriptor: [Myocardial Infarction] this term only |
| #47 | MeSH descriptor: [Myocardial Ischemia] this term only |
| #48 | MeSH descriptor: [Coronary Disease] this term only |
| #49 | MeSH descriptor: [Coronary Artery Bypass] this term only |
| #50 | MeSH descriptor: [Heart Bypass, Left] this term only |
| #51 | MeSH descriptor: [Heart Bypass, Right] this term only |
| #52 | MeSH descriptor: [Myocardial Revascularization] this term only |
| #53 | MeSH descriptor: [Heart Failure] this term only |
| #54 | (coronary OR myocard* OR cardiac* OR heart attack* OR heart infarct* OR angina OR “heart failure” OR heart disease* OR CABG OR PTCA OR ((heart or cardiac) AND stent*) OR “heart bypass” OR postmyocard*):ti,ab,kw |
| #55 | {OR #46-#54} |
| #56 | (rehab*):ti,ab,kw |
| #57 | MeSH descriptor: [Rehabilitation] this term only |
| #58 | #56 OR #57 |
| #59 | #55 AND #58 |
| #60 | #17 AND #28 AND #45 AND #59 |
| #61 | Trials |
