# Supplementary material for: Effectiveness of eHealth Interventions on Moderate-to-Vigorous Intensity Physical Activity Among Patients in Cardiac Rehabilitation: Systematic Review and Meta-analysis
Source: J Med Internet Res. 2023 Mar 29;25:e42845. doi: 10.2196/42845 (PMC10131595; doi:10.2196/42845)
Supplement: Multimedia Appendix 4 [file jmir_v25i1e42845_app4.docx]

**Multimedia Appendix 4**

Study characteristics.

| Author  Year  Country | Study design | Referral diagnosis  Phase of  CR | Group | Age:  mean (SD) | Sample size | Male% | Intervention length | eHealth Component   1. Wearable devices 2. Online web portal 3. Smart phone application 4. Messaging services 5. Telephone calls | Content description of IG or CG | Outcomes   1. MVPA/MPA/VPA 2. CRF 3. WC 4. SBP |
| --- | --- | --- | --- | --- | --- | --- | --- | --- | --- | --- |
| Included in meta-analysis | | | | | | | | | | |
| Hakal et al [40]  2021  Finland | Cluster RCT | MI, CABG, PTCA  Phase II | IG | 59.7 (6.0) | 26 | 81.3% | 48 weeks | 1. A wrist-worn Fitbit Charge HR activity monitor 2. Movendos mCoach internet software 3. NA 4. NA 5. NA | Received an additional distance technology program, set and monitored individual goals and received instructions on how to perform exercises. Also performed PA self-monitoring with a wrist-worn Fitbit Charge HR activity monitor. | 1. MVPA 2. NA 3. NA 4. NA |
|  |  |  | CG | 59.7 (6.0) | 27 |  |  | NA | Received the usual rehabilitation program. |  |
| Reid et al [41]  2021  Canada | Multi-  center  RCT | CHD  Phase III | IG | F: 65.3 (10.3);  M: 63.0 (9.8) | F: 68;  M: 158 | 69.9% | 50 weeks | 1. NA 2. NA 3. NA 4. NA 5. Telephone calls | Received a face-to-face counseling sessions, 5 small-group counseling teleconferences and 3 personal calls from a trained facilitator. | 1. MVPA 2. VO_2_ peak 3. WC 4. SBP |
|  |  |  | CG | F: 66.1 (10.5);  M: 63.2 (9.4) | F: 67;  M: 156 |  |  | NA | Received an updated exercise prescription. |  |
| Engelen et al [42]  2020  Netherlands | Single center RCT | MI  Phase II | IG | 63.3 (10.0) | 103 | 68.3% | 48 weeks | 1. NA 2. “The Vascular View program” 3. NA 4. NA 5. NA | Received access to visit the web-based self-management program called “The Vascular View”, which included 6 modules. Each module comprised 3 or 4 personalized. | 1. MPA, VPA 2. NA 3. NA 4. SBP |
|  |  |  | CG | 63.7 (9.8) | 105 |  |  | NA | Received care as usual. |  |
| Ozemek et al [63]  2020  USA | Quasi-  experimental | MI, CABG, PCI, heart valve repair, stable angina  Phase III | IG1 | 62.2 (9.1) | 29 | 77.8% | 12 weeks | 1. PA monitor (Actigraph   GT3X and Lifecorder PLUS)   1. NA 2. NA 3. Motivational messaging 4. NA | PF: Received a weekly goal to increase PA by 10% of baseline values and were asked to record step counts 4 times each day to obtain immediate feedback via pedometer on daily progress toward goal. | 1. MVPA 2. NA 3. NA 4. NA |
|  |  |  | IG2 | 62.0 (12.8) | 25 |  |  |  | MM: Received a weekly newsletter containing both cognitive and behavioral information to encourage PA and received a stage-specific booklet on a weekly basis. |  |
|  |  |  | IG3 | 61.1 (9.8) | 24 |  |  |  | PF + MM: Received the same PA step goals as the PF group and received newsletters relative to whether or not accomplished PA goal for the week. |  |
|  |  |  | CG | 60.3 (11.5) | 21 |  |  | NA | Received phase III CR program and recommendations to perform 30 to 50 min of home exercise on ≥ 2 non-CR days. |  |
| Claes et al [43]  2020  Belgium  Ireland | Multi-  center RCT (pilot) | CVD  Phase III | IG | 61.7 (14.5) | 60 | 81.7% | 24 weeks | 1. HR monitor 2. “Physical Activity Toward   Health (PATHway) system”   1. NA 2. NA 3. NA | Received access to visit PATHway-I system, which included 5 different modules. Also received a Microsoft Band 2 heart rate monitor to achieve the prescribed target HR zone and received text-based resource content about self-monitoring, feedback on performance, goal-setting and rewards. | 1. MVPA 2. VO_2_ peak 3. NA 4. SBP |
|  |  |  | CG | 59.6 (13.2) | 60 |  |  | NA | Received verbal advice on how to best maintain PA and a heart-healthy lifestyle. |  |

| Author  Year  Country | Study design | Referral diagnosis  Phase of  CR | Group | Age:  mean (SD) | Sample size | Male% | Intervention length | eHealth Component   1. Wearable devices 2. Online web portal 3. Smart phone application 4. Messaging services 5. Telephone calls | Content description of IG or CG | Outcomes   1. MVPA/MPA/VPA 2. CRF 3. WC 4. SBP |
| --- | --- | --- | --- | --- | --- | --- | --- | --- | --- | --- |
| Included in meta-analysis | | | | | | | | | | |
| Barnason et al [44]  2019  USA | Single center RCT | After CABS or PCI | IG | 63 (9.3) | 22 | 69.8% | 12 weeks | 1. NA 2. The Viterion® telehealth device 3. NA 4. NA 5. Telephone coaching | Received weight management intervention (WMI) plus CR. WMI included education component, skill component and telephone coaching component. The WMI education component included written target caloric goals, diet portion guidelines and a handbook for use of portion control guidelines. The WMI skill component included a 6-week telehealth component delivered by the Viterion® device, that included 6 modules with 36 telehealth sessions. The WMI telephone coaching sessions were delivered by the research nurse at week 9 and 12. | 1. Moderate or more intense 2. NA 3. NA 4. NA |
|  |  |  | CG |  | 21 |  |  | NA | Received the outpatient CR sessions. |  |
| Maddison et al [45]  2019  New Zealand | Multi-  center RCT | CHD  Phase II | IG | 61.0 (13.2) | 68 | 85.8% | 12 weeks | 1. Chest-worn wearable sensor 2. REMOTE-CR Platform 3. Bespoke smart phone and web application 4. Short message service 5. NA | Received individualized exercise prescription, exercise monitoring and coaching (via a chest-worn wearable sensor) to set individualized goals and review automated goal achievement feedback (via the smartphone and web apps) plus theory-based behavioral strategies to promote exercise and habitual PA (via short message services), delivered via a bespoke telerehabilitation platform (REMOTE-CR platform). | 1. MPA, VPA 2. VO_2_ peak 3. WC 4. SBP |
|  |  |  | CG | 61.5 (12.2) | 72 |  |  | NA | Received supervised exercise delivered by clinical exercise  physiologists in CR clinics. |  |
| Prince et al [46]  2018  Canada | Single center RCT (pilot) | CAD  Phase II | IG | 62.4 (10.7) | 19 | 60.5% | 8 weeks | 1. VTAP monitor 2. NA 3. NA 4. NA 5. NA | Received a VTAP monitor, provided real-time feedback via alerts (‘buzzing’/mild vibration) once had been sedentary for 30 consecutive min and required 2 min of standing/movement to reset. | 1. MVPA 2. VO_2_ peak 3. WC 4. SBP |
|  |  |  | CG | 61.5 (9.7) | 21 |  |  | NA | Received regular CR included supervised twice weekly exercise sessions over an 8-week period at the Prevention and Rehabilitation Center. |  |
| Duscha et al [47]  2018  USA | Single center RCT (pilot) | CVD  Phase III | IG | 59.9 (8.1) | 16 | 76% | 12 weeks | 1. Fitbit activity trackers 2. NA 3. “Vida Health” 4. emails and text messaging 5. Telephone calls | Received a Fitbit activity tracker, downloaded the “Vida Health” and received an initial personalized exercise prescription, which was shared with the Vida health coaches to support the exercise portion of the intervention during weekly coaching sessions via telephone calls better. In addition, coaches sent educational material via email and text messages. | 1. MVPA 2. VO_2_ peak 3. NA 4. NA |
|  |  |  | CG | 66.5 (7.2) | 9 |  |  | NA | Received encouragement to follow the guidance provided by physician. |  |
| Salvi et al [48]  2018  Spain | Multi-  center RCT | MI  Phase III | IG | 59 (14) | 8 | 89% | 8 weeks | 1. A wearable sensor 2. A tablet PC with 5 sections 3. A smartphone APP 4. NA 5. NA | Received the GEx system, which is composed of three main parts: the mobile station, for monitoring physical exercise; the patient station, which is responsible for delivering educational contents to the user; and the professional station, a web-based application used by doctors. | 1. MVPA 2. NA 3. NA 4. NA |
|  |  |  | CG |  | 7 |  |  | NA | Received standard rehabilitation according to the national procedures. |  |

| Author  Year  Country | Study design | Referral diagnosis  Phase of  CR | Group | Age:  mean (SD) | Sample size | Male% | Intervention length | eHealth Component   1. Wearable devices 2. Online web portal 3. Smart phone application 4. Messaging services 5. Telephone calls | Content description of IG or CG | Outcomes   1. MVPA/MPA/VPA 2. CRF 3. WC 4. SBP |
| --- | --- | --- | --- | --- | --- | --- | --- | --- | --- | --- |
| Included in meta-analysis | | | | | | | | | | |
| Young et al [49]  2016  USA | Single center RCT | HF  Phase II | IG | 68.7 (11.8) | 51 | 36% | 12 weeks | 1. NA 2. NA 3. NA 4. NA 5. Telephone calls | Received a one-on-one in-hospital self-management training session and post-discharge reinforcement sessions (twice a week for the first 2 weeks, once a week for weeks 3-6, and every other week for weeks 7-12) delivered by telephone. | 1. Moderate or more intense 2. NA 3. NA 4. NA |
|  |  |  | CG | 71.8 (12.6) | 49 |  |  | NA | Received only usual care, the standard discharge teaching for HF that includes written and verbal information about HF self-care and scheduled follow-up doctor appointments. |  |
| Alsaleh et al [50]  2016  Jordan | Multi-  center RCT | CHD  Phase II | IG | 59.5 (9.9) | 66 | 53.8% | 24 weeks | 1. NA 2. NA 3. NA 4. Text messaging 5. Telephone calls | Received a face-to-face consultation lasting 20-30 min and 6  telephone call-based consultations by a cardiac nurse (15-20 min once time/month). Received 18 reminder text messages, which informed by the TPB and based on reminding to perform and maintain the required level of PA and encouraging to address barriers to PA. | 1. MPA 2. NA 3. NA 4. SBP |
|  |  |  | CG | 61.4 (9.7) | 79 |  |  | NA | Received usual care from physicians, which consisted of general (rather than tailored) advice about the benefits of PA and instructions to engage in MPA, such as brisk walking. |  |
| Frederix et al [51]  2015  Belgium | Multi-  center RCT | CAD, CHF  Phase III | IG | 61 (9) | 69 | 82% | 24 weeks | 1. Motion sensor (Yorbody accelerometer) 2. NA 3. NA 4. email and SMS text messaging 5. NA | Received an Internet-based, comprehensive telerehabilitation program. Received PA telemonitoring via a motion sensor and received encourage feedback by a semiautomatic tele-coaching system via emails and SMS text messages. | 1. MPA, VPA 2. VO_2_ peak 3. NA 4. SBP |
|  |  |  | CG | 61 (8) | 70 |  |  | NA | Received a conventional CBCR program, including 45 pluri-disciplinary rehabilitation sessions with at least 2 exercise training sessions per week. |  |
| Kaminsky et al [64]  2013  USA | Quasi-  experimental | MI, CABG,  PCI, heart  valve repair,  stable angina,  Phase III | IG | 53.3 (8.1) | 10 | 77.8% | 7 weeks | 1. Pedometer 2. NA 3. NA 4. NA 5. NA | Received individualized daily step count goals to increase by 10% of baseline steps/day for weeks 2-8 and were instructed to obtain pedometer feedback by recording step counts at lunch, dinner, and bedtime to encourage compliance with the daily step count goals. | 1. MVPA 2. NA 3. NA 4. NA |
|  |  |  | CG | 53.3 (8.1) | 8 |  |  | NA | Received the maintenance CR program and recommendation to obtain a minimum of 30-40 min/day MVPA on days did not attend CR. |  |
| Guiraud et al [52]  2012  France | Single center RCT | CAD, HF  Phase III | IG | 54.5 (12.6) | 19 | 82.8% | 8 weeks | 1. Accelerometer 2. NA 3. NA 4. NA 5. Telephone calls | Received an accelerometer to measure and record PA. Also received telephone calls every 15 days by kinesiologist to motivate PA, which were given standard feedback on the amount of PA performed. | 1. MPA 2. NA 3. NA 4. NA |
|  |  |  | CG | 62.9 (10.7) | 10 |  |  | NA | Received no telephone calls and were only allowed to wear the accelerometer during the 8th week of the intervention. |  |
| Reid et al [53]  2012  Canada | Multi-  center RCT | ACS  Phase II | IG | 56.7 (9.0) | 115 | 84.3% | 24 weeks | 1. NA 2. “CardioFit” system 3. NA 4. Emails 5. NA | Received a personally tailored PA plan generated by an exercise specialist via the CardioFit system and were asked to log daily activity on the CardioFit website and complete a series of five online tutorials following hospital discharge. Received emails from the exercise specialist providing motivational feedback on their progress. | 1. MVPA 2. NA 3. NA 4. NA |
|  |  |  | CG | 56.0 (9.0) | 108 |  |  | NA | Received PA guidance from attending cardiologist and an education booklet. |  |

| Author  Year  Country | Study design | Referral diagnosis  Phase of  CR | Group | Age:  mean (SD) | Sample size | Male% | Intervention length | eHealth Component   1. Wearable devices 2. Online web portal 3. Smart phone application 4. Messaging services 5. Telephone calls | Content description of IG or CG | Outcomes   1. MVPA/MPA/VPA 2. CRF 3. WC 4. SBP |
| --- | --- | --- | --- | --- | --- | --- | --- | --- | --- | --- |
| Included in meta-analysis | | | | | | | | | | |
| Reid et al [54]  2012  Canada | Single center RCT | ACS  Phase II | IG | 59.5 (9.9) | 69 | 73% | 52 weeks | 1. NA 2. NA 3. NA 4. NA 5. Telephone calls | Received 8 motivational counselling via telephone by a specially trained physiotherapist. | 1. MVPA 2. NA 3. NA 4. NA |
|  |  |  | CG | 61.4 (9.7) | 72 |  |  | NA | Received a Cardiology Discharge Book. |  |
| Barnason et al [55]  2009  USA | Multi-  center RCT | CABG  Phase II | IG | 71.21 (4.91) | 81 | 82.8% | 6 weeks | 1. NA 2. NA 3. “Health Buddy ®” 4. NA 5. NA | Received a six-week Symptom Management intervention which delivered 42 daily sessions via the Health Buddy ® teleHealth device and provided subjects with strategies designed to address commonly occurring symptoms experienced after recovery from CABG. | 1. Moderate, hard and very hard intensity PA 2. NA 3. NA 4. NA |
|  |  |  | CG |  | 99 |  |  | NA | Received usual care. |  |

| Author  Year  Country | Study design | Referral diagnosis  Phase of  CR | Group | Age:  mean (SD) | Sample size | Male% | Intervention length | eHealth Component   1. Wearable devices 2. Online web portal 3. Smart phone application 4. Messaging services 5. Telephone calls | Content description of IG or CG | Outcomes   1. MVPA/MPA/VPA 2. CRF 3. WC 4. SBP |
| --- | --- | --- | --- | --- | --- | --- | --- | --- | --- | --- |
| Not included in meta-analysis | | | | | | | | | | |
| Pinto et al [65]  2022  Portugal | Pre-posttest | CVD  Phase III | IG | 62.6 (8.9) | 116 | 81.9% | 12 weeks | 1. NA 2. An online platform 3. NA 4. NA 5. NA | Received a home-based cardiac rehabilitation program, including an (1) online exercise training sessions, (2) online educational sessions, and (3) psychological group sessions. | 1. MVPA, MPA and VPA 2. NA 3. NA 4. SBP |
| Legler et al [66]  2020  USA | Pre-posttest | ACS Phase II | IG | 63.4 (9.7) | 40 | 75.6% | 2 weeks | 1. NA 2. NA 3. NA 4. Text message 5. NA | Received a single text message per day for 14 days, alternating between messages focusing on positive psychology content and messages focusing on specific suggestions for physical activity each day. | 1. MPA 2. NA 3. NA 4. NA |
| Freene et al [67]  2020  Australia | Pre-  posttest (feasibility) | CHD  Phase III | IG | 54 (13) | 20 | 85% | 16 weeks | 1. A wrist-worn Fitbit Flex 2. NA 3. “Vire” app (“ToDo-CR” program) and Fitbit app 4. NA 5. NA | Received the ToDo-CR program which was personalized and consists of different types of Do’ s delivered through the smartphone app via push notifications. The Do’ s are small actionable and achievable goals based on the individual’s data. | 1. MVPA 2. 6MWT 3. NA 4. SBP |
| Sengupta et al [68]  2020  USA | Pre-posttest (usability) | CHD  Phase II | IG | 64.4 (6.3) | 10 | 0 | 12 weeks | 1. Smartwatch 2. NA 3. “HerBeat” 4. NA 5. NA | Received a smartwatch and a smartphone with an app called “HerBeat”, which included 4 features: (1) goal setting, (2) progress, (3) ecological momentary assessment surveys, and (4) videos. | 1. MPA 2. NA 3. NA 4. NA |
| Chan et al [56]  2022  China | Multi-center RCT | CHD  Phase II | IG | 59.8 (6.4) | 70 | 71.2% | 12 weeks | 1. NA 2. NA 3. ZTExApp 4. ZTEx picture e-messages 5. NA | Received Zero-time Exercise intervention, including a 15-min individual face-to-face session included ZTEx and a smartphone ZTExApp; and a package of 28 anti-inertia reminder ZTEx picture e-message. | 1. MPA, VPA 2. NA 3. WC 4. NA |
|  |  |  | CG | 59.8 (6.8) | 69 |  |  |  | Received healthy eating and breathing exercise information, including a 15-min individual face-to-face session included healthy eating and breathing exercise; and a package of 28 healthy eating and breathing exercise picture e-messages |  |
| Pate et al [57]  2021  USA | Single center RCT | ASCVD  Phase III | IG1 | 57.4 (10.6) | 99 | 30.4% | 16 weeks | 1. Fitbit Alta or Fitbit Inspire 2. NA 3. NA 4. Text message 5. NA | Each of the 4 intervention groups received an assigned (or choice) and immediate (or gradual) daily step goal and a gamification intervention based on the principles of behavioral economics via text message.  IG1: Received gamification with assigned and gradual goals.  IG2: Received gamification with assigned and immediate goals.  IG3: Received gamification with choice and gradual goals.  IG4: Received gamification with choice and immediate goals. | 1. MVPA 2. NA 3. NA 4. NA |
|  |  |  | IG2 | 57.5 (10.8) | 99 |  |  |  |  |  |
|  |  |  | IG3 | 58.7 (11.8) | 106 |  |  |  |  |  |
|  |  |  | IG4 | 58 (10.9) | 100 |  |  |  |  |  |
|  |  |  | CG | 61.1 (9.3) | 96 |  |  |  | Participants were asked to use the wearable device but did not establish daily step goals and received daily feedback via text message on the previous day’s step count. |  |
| Kayser et al [58]  2019  Canada | Multi-  center RCT | ACS  Phase II | IG | 52.9 (9.3) | 22 | 88.3% | 12 weeks | 1. NA 2. TAVIE en m@rche 3. NA 4. NA 5. NA | Received a web-based tailored intervention, TAVIE en m@rche, consisted of intervention content in pre-recorded videos to gradually increase moderate-intensity walking behavior, up to 150 weekly minutes. | 1. MVPA 2. NA 3. NA 4. NA |
|  |  |  | CG | 58.6 (9.6) | 15 |  |  | NA | Received a list of hyperlinks on one unique webpage of four public websites that contained information in text format on walking. |  |

| Author  Year  Country | Study design | Referral diagnosis  Phase of  CR | Group | Age:  mean (SD) | Sample size | Male% | Intervention length | eHealth Component   1. Wearable devices 2. Online web portal 3. Smart phone application 4. Messaging services 5. Telephone calls | Content description of IG or CG | Outcomes   1. MVPA/MPA/VPA 2. CRF 3. WC 4. SBP |
| --- | --- | --- | --- | --- | --- | --- | --- | --- | --- | --- |
| Not included in meta-analysis | | | | | | | | | | |
| Antypas et al [59]  2014  Norway | Single center RCT | CVD  Phase II | IG | 59.5 (8.8) | 7 | 77.6% | 12 weeks | 1. NA 2. “Don’t give up” 3. NA 4. NA 5. NA | Received access to the same functionality as the CG as well as access to tailored intervention content. Asked to plan training activities or set weekly goals then received feedback in the form of a simple graph on the website regarding the achievement of goals. | 1. MPA, VPA 2. NA 3. NA 4. NA |
|  |  |  | CG | 58.8 (9.4) | 12 |  |  | NA | Received access to the basic Internet-based intervention “Don’t give up”, which contained general information about CVD and self-management, as well as access to an online discussion forum. Enabled to plan training activities, but were not prompted to do it and received no feedback. |  |
| Avila et al [60]  2018  Belgium | Single center RCT | CAD  Phase III | IG | 58.6 (13) | 24 | 88.9% | 12 weeks | 1. HR monitor 2. NA 3. Garmin Platform 4. Emails 5. Telephone calls | Received an individualized aerobic exercise prescription recommending at least 150 min of exercise per week in their home environment, received instructions on how to use the HR monitor and how to upload exercise data to the Garmin platform, and according to preferences received feedback via phone or email once a week. | 1. MVPA 2. VO_2_ peak 3. NA 4. SBP |
|  |  |  | CG1 | 61.9 (7.3) | 28 |  |  | NA | Center-based: Received exercise program at the outpatient clinic of UZ Leuven under the direct supervision of physiotherapists. |  |
|  |  |  | CG2 | 61.7 (7.7) | 26 |  |  | NA | Control: Received usual care including the standard advice to remain physically active. |  |
| Peydró et al [61]  2022  Spain | Multi-center RCT | ACS  Phase III | IG | 57.5 (9.0) | 31 | 91.5% | 40 weeks | 1. NA 2. A web platform 3. A cardiac telerehabilitation system called *Cardioplan* 4. NA 5. NA | Participants had access to a password-protected webpage that allowed personalized healthcare and tracking of patient adherence to recommendations. And used the smartphone application to schedule daily exercise sessions and record subjective general condition, vital signs, and medication adherence; the app's exercise module also tracked and recorded every exercise session and provided warm-up and stretching videos, a virtual education classroom, and suggested websites. | 1. MPA, VPA 2. VO_2_ peak 3. NA 4. NA |
|  |  |  | CG | 54.7 (9.9) | 28 |  |  | NA | Received 2 months of treatment with 16 sessions of supervised exercise. The physical activity consisted of routine workouts and aerobic cycling training. |  |
| Devi et al [62]  2014  UK | Single center RCT | Stable  Angina  Phase II | IG | 66.27 (8.35) | 35 | 74.5% | 6 weeks | 1. NA 2. “ActivateYourHeart” 3. NA 4. NA 5. NA | Received a Web-based rehabilitation program (“ActivateYourHeart”), contained information about the secondary prevention of CHD and set each user goals, and could initiate contact with CR nurses for advice and support via an online email link or by joining a scheduled synchronized chat room held on a weekly basis. | 1. MPA 2. NA 3. NA 4. SBP |
|  |  |  | CG | 66.20 (10.06) | 40 |  |  | NA | Received treatment as usual from general practitioners. |  |
