# Supplementary material for: Effectiveness of eHealth Interventions on Moderate-to-Vigorous Intensity Physical Activity Among Patients in Cardiac Rehabilitation: Systematic Review and Meta-analysis
Source: J Med Internet Res. 2023 Mar 29;25:e42845. doi: 10.2196/42845 (PMC10131595; doi:10.2196/42845)
Supplement: Multimedia Appendix 5 [file jmir_v25i1e42845_app5.docx]

**Multimedia Appendix 5**

Moderate-to-vigorous intensity physical activity, moderate-intensity physical activity, and vigorous-intensity physical activity outcomes data from included studies.

| *Included in the meta-analysis, data presented as mean±SD* | | | | | | | | | | | | |  |  |
| --- | --- | --- | --- | --- | --- | --- | --- | --- | --- | --- | --- | --- | --- | --- |
| Studies | MVPA measurement  tool | MVPA measurement method | Units of measurement | IG | IG: Baseline MVPA | | IG: Follow-up MVPA | | CG: Baseline MVPA | | CG: Follow-up MVPA | | |  |
|  |  |  |  |  | Mean | SD | Mean | SD | Mean | SD | Mean | SD | |  |
| Reid et al [41], 2021 | Actigraph GT3X+ accelerometer | Objective | Minutes/week | NA | F: 272.9 | F: 154.7 | F: 248.1 | F: 160.6 | F: 238.6 | F: 167.1 | F: 216.9 | F: 179.8 | | |
|  |  |  |  |  | M: 314.1 | M: 195.9 | M: 282.9 | M: 206.9 | M: 331.0 | M: 207.4 | M: 284.8 | M: 202.3 | | |
| Engelen et al [42], 2020 | IPAQ, short version | Self-reported | Minutes/week (moderate) | NA | 385 | 323 | 385 | 276 | 362 | 354 | 340 | 300 | | |
|  |  |  | Minutes/week (vigorous) | NA | 185 | 211 | 182 | 217 | 117 | 174 | 161 | 215 | | |
| Ozemek et al [63], 2020 | Actigraph GT3X | Objective | Minutes/day | PF | 29 | 35.9 | 38.5 | 43 | 21.3 | 23.5 | 24.6 | 19.7 | | |
|  |  |  |  | MM | 33.1 | 59.7 | 34.9 | 55.8 |  |  |  |  | | |
|  |  |  |  | PF+MM | 19.2 | 17.5 | 22.3 | 18.4 |  |  |  |  | | |
| Claes et al [43], 2020 | Actigraph GT9X Link | Objective | Minutes/day | NA | 127 | 57.9 | 141 | 69.1 | 146 | 65.9 | 143 | 70.6 | | |
| Barnason et al [44], 2019 | Actigraph® GT3X | Objective | Minutes/day (moderate or more intense) | NA | NA | NA | 14.4 | 16.1 | NA | NA | 11 | 7.9 | | |
| Maddison et al [45], 2019 | Actigraph uniaxial accelerometer | Objective | Minutes/day (moderate) | NA | 24.6 | 20.6 | 25.3 | 17.6 | 24.3 | 16.3 | 30.6 | 18.4 | | |
|  |  |  | Minutes/day (vigorous) |  | 0.4 | 1.3 | 1.4 | 3.9 | 0.3 | 1.8 | 0.8 | 3.3 | | |
| Prince et al [46], 2018 | ActivPAL3 activity monitor | Objective | Minutes/day | NA | 22 | 15.6 | 32.6 | 24.3 | 16.4 | 15.6 | 25.2 | 15.7 | | |
|  | IPAQ | Self-reported |  |  | 19.7 | 21.9 | 76.1 | 60.9 | 4.3 | 9.3 | 57.8 | 35.3 | | |
| Duscha et al [47], 2018 | Fitbit activity trackers | Objective | Minutes/week | NA | 138 | 113 | 159 | 156 | 111 | 87 | 65 | 64 | | |
| Salvi et al [48], 2018 | A custom questionnaire | Self-reported | Minutes/week | NA | 138.85 | 53.39 | 161.88 | 77.69 | 146.28 | 60.04 | 140 | 54.27 | | |
| Young et al [49], 2016 | ActiGraph | Objective | Minutes/day (moderate or more intense) | NA | NA | NA | 5.8 | 8.8 | NA | NA | 3.3 | 10.5 | | |
| Alsaleh et al [50], 2016 | IPAQ, short version | Self-reported | Minutes/week (moderate) | NA | 21.14 | 44.95 | 36.67 | 109.76 | 20.13 | 62.50 | 16.46 | 64 | | |
| Frederix et al [51], 2015 | IPAQ | Self-reported | MET-min/week (moderate) | NA | 362 | 703 | 619 | 1049 | 592 | 1084 | 405 | 757 | | |
|  |  |  | MET-min/week (vigorous) |  | 302 | 729 | 648 | 1953 | 369 | 827 | 190 | 537 | | |
| Kaminsky et al [64], 2013 | NL-1000 pedometers | Objective | Minutes/day | NA | 10.2 | 8.0 | 17.7 | 10.5 | 8.5 | 8.0 | 7.7 | 10.5 | | |
| Guiraud et al [52], 2012 | MyWellness Key accelerometer | Objective | Minutes/week (moderate) | NA | 70.1 | 32.4 | 137.2 | 87.5 | 52.5 | 33.6 | 45.7 | 43.4 | | |
| Reid et al [53], 2012 | Godin Leisure Time Exercise Questionnaire | Self-reported | Minutes/week | NA | NA | NA | 201.0 | 153.2 | NA | NA | 163.4 | 151.3 | | |
| Reid et al [54], 2012 | Godin Leisure Time Exercise Questionnaire version 10 | Self-reported | Minutes/week | NA | 80.5 | 150.2 | 233.8 | 189.5 | 102.6 | 207.1 | 168.8 | 176.6 | | |
| Barnason et al [55], 2009 | Physical Activity and Exercise Diary | Self-reported | Minutes/day (moderate, hard, and very hard) | NA | NA | NA | 145.4 | 118.8 | NA | NA | 126.9 | 195.7 | | |

| *Included in the meta-analysis, data presented as MD* | | | | | | | | |
| --- | --- | --- | --- | --- | --- | --- | --- | --- |
| Studies | MVPA measurement tool | MVPA measurement method | Units of measurement | IG | Effect of the Intervention | | Effect of the Control | |
|  |  |  |  |  | MD | SD of the Difference | MD | SD of the Difference |
| Hakal et al [40], 2021 | A hip-worn Fitbit Zip accelerometer | Objective | Minutes/week | NA | 64.1 | 355.17 | -6.8 | 179.23 |
|  | IPAQ | Self-reported |  |  | -76.4 | 772.82 | 215.9 | 913.58 |

| *Not included in the meta-analysis, no CG* | | | | | | | | | |  |
| --- | --- | --- | --- | --- | --- | --- | --- | --- | --- | --- |
| Studies | MVPA measurement tool | MVPA measurement method | Units of measurement | IG | IG: Baseline MVPA | | | IG: Follow-up MVPA | | |
|  |  |  |  |  | Mean | SD | Mean | | SD |  |
| Pinto et al [65], 2022 | IPAQ, short form | Self-reported | Minutes/week | NA | 230 | 198 | 393 | | 378 |  |
|  |  |  | Minutes/week (moderate) |  | 189 | 158 | 301 | | 321 |  |
|  |  |  | Minutes/week (vigorous) |  | 48 | 86 | 107 | | 157 |  |
| Legler et al [66], 2020 | A structured, validated two-item measure | Self-reported | Minutes/week (moderate) | NA | 261.5 | 265 | 366 | | 519 |  |
| Freene et al [67], 2020 | A triaxial commercial accelerometer | Objective | Minutes/day | NA | 74 | 23 | 77 | | 31 |  |
| Sengupta et al [68], 2020 | IPAQ, short version | Self-reported | Minutes/day (moderate) | NA | 35.7 | 35.3 | 63.1 | | 52.8 |  |

| *Not included in the meta-analysis, CG with eHealth component* | | | | | | | | | | | | | |
| --- | --- | --- | --- | --- | --- | --- | --- | --- | --- | --- | --- | --- | --- |
| Studies | MVPA Measurement tool | MVPA measurement method | Units of  measurement | IG | IG: Baseline MVPA | | IG: Follow-up MVPA | | CG: Baseline MVPA | | CG: Follow-up MVPA | |  |
|  |  |  |  |  | Mean | SD | Mean | SD | Mean | SD | Mean | SD |  |
| Chan et al [56], 2022 | IPAQ, Chinese version | Self-reported | Minutes/week (moderate) | NA | 55.1 | NA | 112.9 | NA | 44.6 | NA | 83.6 | NA |  |
|  |  |  | Minutes/week (vigorous) |  | 5.9 | NA | 2.7 | NA | 4.8 | NA | 7.4 | NA |  |
| Pate et al [57], 2021 | Fitbit Alta or Fitbit Inspire | Objective | Minutes/day | IG1 | 5.8 | 7.6 | 9.2 | 8.3 | 6.8 | 8.3 | 7.6 | 9 |  |
|  |  |  |  | IG2 | 5.8 | 6.9 | 9.9 | 9.6 |  |  |  |  |  |
|  |  |  |  | IG3 | 5.3 | 6.5 | 9.1 | 10.4 |  |  |  |  |  |
|  |  |  |  | IG4 | 7 | 7.5 | 11.9 | 12.9 |  |  |  |  |  |

| *Not included in the meta-analysis, CG with eHealth component* | | | | | | | | | | |
| --- | --- | --- | --- | --- | --- | --- | --- | --- | --- | --- |
| Studies | MVPA measurement tool | MVPA measurement method | Units of measurement | IG | Effect of the Intervention | | | Effect of the Control | | |
|  |  |  |  |  | MD | SD of the Difference | MD | | SD of the Difference |  |
| Kayser et al [58], 2019 | IPAQ, short version | Self-reported | Minutes/week | NA | 1260.4 | 2835.8 | -203.9 | | 2843.9 |  |

| *Not included in the meta-analysis, CG with eHealth component* | | | | | | | | | | | | | | | | | | | | | | |
| --- | --- | --- | --- | --- | --- | --- | --- | --- | --- | --- | --- | --- | --- | --- | --- | --- | --- | --- | --- | --- | --- | --- |
| Studies | MVPA measurement tool | MVPA measurement method | Units of measurement | IG | IG: Baseline MVPA | | | | IG: Follow-up MVPA | | | | CG: Baseline MVPA | | | | CG: Follow-up MVPA | | | |  |  |
|  |  |  |  |  | Median | | IQR | | Median | | IQR | | Median | | IQR | | Median | | IQR | |  |  |
| Antypas et al [59], 2014 | IPAQ | Self-reported | MET-min/week (moderate) | NA | 1440.0 | | 2400.0 | | 1440.0 | | 2000.0 | | 930.0 | | 1320.0 | | 480.0 | | 1080.0 | |  |  |
|  |  |  | MET-min/week (vigorous) |  | | 3240.0 | | 4260.0 | | 2300.0 | | 1824.0 | | 2400.0 | | 2802.0 | | 0 | | 1920.0 | |  |

| *Not included in the meta-analysis, no SD reported* | | | | | | | | | | | | | |
| --- | --- | --- | --- | --- | --- | --- | --- | --- | --- | --- | --- | --- | --- |
| Studies | MVPA Measurement tool | MVPA measurement method | Units of  measurement | IG | IG: Baseline MVPA | | IG: Follow-up MVPA | | CG: Baseline MVPA | | CG: Follow-up MVPA | |  |
|  |  |  |  |  | Mean | Range | Mean | Range | Mean | Range | Mean | Range |  |
| Avila et al [60], 2018 | A Sensewear Mini Armband | Objective | Minutes/day | NA | 145 | 34-299 | 141 | 51-259 | 146 | 28-417 | 134 | 29-366 |  |
|  |  |  | Minutes/day (moderate) |  | 136 | 34-238 | 134 | 49-241 | 140 | 28-391 | 128 | 27-348 |  |
|  |  |  | Minutes/day (vigorous) |  | 8 | 0-33 | 7 | 0-24 | 6 | 0-26 | 6 | 0-24 |  |

| Not included in the meta-analysis, data presented as median | | | | | | | | | | | | |  |
| --- | --- | --- | --- | --- | --- | --- | --- | --- | --- | --- | --- | --- | --- |
| Studies | MVPA  measurement tool | MVPA measurement method | Units of  measurement | IG | IG: Baseline MVPA | | IG: Follow-up MVPA | | CG: Baseline MVPA | | CG: Follow-up MVPA | |  |
|  |  |  |  |  | Median | IQR | Median | IQR | Median | IQR | Median | IQR | |
| Peydró et al [61], 2022 | IPAQ | Self-reported | MET-min/week (moderate) | NA | 140 | 0-720 | 580 | 240-2400 | 440 | 0-2700 | 540 | 0-1020 | |
|  |  |  | MET-min/week (vigorous) |  | 0 | 0-320 | 620 | 0-2400 | 0 | 0-240 | 0 | 0-1960 | |
| Devi et al [62], 2014 | Sensewear Pro 3 accelerometer | Objective | Minutes/day (moderate) | NA | 43.5 | 43.00 | 48.5 | 50 | 55.50 | 96.25 | 47.75 | 61.38 | |
