# Supplementary material for: Effectiveness of eHealth Interventions on Moderate-to-Vigorous Intensity Physical Activity Among Patients in Cardiac Rehabilitation: Systematic Review and Meta-analysis
Source: J Med Internet Res. 2023 Mar 29;25:e42845. doi: 10.2196/42845 (PMC10131595; doi:10.2196/42845)
Supplement: Multimedia Appendix 6 [file jmir_v25i1e42845_app6.docx]

**Multimedia Appendix 6**

Summary of primary and secondary outcomes in the included studies.

| Outcomes | RCTs | | Quasi-experimental studies | | Pre-posttest studies | |
| --- | --- | --- | --- | --- | --- | --- |
|  | Study | *P* value | Study | *P* value | Study | *P* value |
| **MVPA (n=17)** | | | | | | |
|  | Hakal et al [40] ^d^, 2021 | *P*>.05 | Ozemek et al [63] ^a^, 2020 | *P*=.08 | Pinto et al [65] ^b^, 2022 | *P*=.001 |
|  | Reid et al [41] ^d,a^, 2021 | F: *P*=.998,  M: *P*=.557 | Kaminsky et al [64] ^b^, 2013 | *P*<.05 | Freene et al [67] ^e^, 2020 | NA |
|  | Claes et al [43]^c^, 2020 | *P*=.04 |  |  |  |  |
|  | Barnason et al [44] ^a^, 2019 | NA |  |  |  |  |
|  | Duscha et al [47] ^c^, 2018 | *P*≤.05 |  |  |  |  |
|  | Salvi et al [48] ^e^, 2018 | NA |  |  |  |  |
|  | Young et al [49] ^a^, 2016 | NA |  |  |  |  |
|  | Reid et al [53] ^e^, 2012 | NA |  |  |  |  |
|  | Reid et al [54] ^e^, 2012 | NA |  |  |  |  |
|  | Barnason et al [55] ^c^, 2009 | *P*<.01 |  |  |  |  |
|  | Pate et al [57] ^c^, 2021 | I1: *P*=.05,  I2: *P*=.008,  I3: *P*=.03,  I4: *P*<.001 |  |  |  |  |
|  | Kayser et al [58] ^d^, 2019 | *P*=.13 |  |  |  |  |
|  | Prince et al [46] ^d,d^, 2018 | O: *P*=.587,  S: *P*=.134 |  |  |  |  |
|  | Avila et al [60] ^d^, 2018 | *P*=.47 |  |  |  |  |
| **MPA (n=14)** | | | | | | |
|  | Engelen et al [42] ^d^, 2020 | *P*=.79 |  |  | Pinto et al [65] ^b^, 2022 | *P*=.001 |
|  | Maddison et al [45] ^a^, 2019 | *P*>.05 |  |  | Legler et al [66] ^a^, 2020 | *P*=.15 |
|  | Alsaleh et al [50] ^c^, 2016 | *P*<.05 |  |  | Sengupta et al [68] ^d^, 2020 | *P*>.05 |
|  | Frederix et al [51] ^e^, 2015 | NA |  |  |  |  |
|  | Guiraud et al [52] ^c^, 2012 | *P*=.002 |  |  |  |  |
|  | Chan et al [56] ^a^, 2022 | *P*=.37 |  |  |  |  |
|  | Antypas et al [59] ^d^, 2014 | *P*>.05 |  |  |  |  |
|  | Avila et al [60] ^d^, 2018 | *P*=.62 |  |  |  |  |
|  | Peydró et al [61] ^c^, 2022 | *P*=.039 |  |  |  |  |
|  | Devi et al [62] ^c^, 2014 | *P*=.01 |  |  |  |  |
| **VPA (n=8)** | | | | | | |
|  | Engelen et al [42] ^d^, 2020 | *P*=.23 |  |  | Pinto et al [65] ^b^, 2022 | *P*=.001 |
|  | Maddison et al [45] ^d^, 2019 | *P*>.05 |  |  |  |  |
|  | Frederix et al [51] ^e^, 2015 | NA |  |  |  |  |
|  | Chan et al [56] ^a^, 2022 | *P*=.23 |  |  |  |  |
|  | Antypas et al [59] ^d^, 2014 | *P*>.05 |  |  |  |  |
|  | Avila et al [60] ^d^, 2018 | *P*=.59 |  |  |  |  |
|  | Peydró et al [61] ^a^, 2022 | *P*=.484 |  |  |  |  |
| **CRF (n=8)** | | | | | | |
|  | Reid et al [41] ^d,a^, 2021 | F: *P*=.323,  M: *P*=.637 |  |  | Freene et al [67] ^b^, 2020 | *P*<.001 |
|  | Claes et al [43] ^a^, 2020 | *P*=.64 |  |  |  |  |
|  | Maddison et al [45] ^d^, 2019 | *P*>.05 |  |  |  |  |
|  | Prince et al [46] ^d^, 2018 | *P*=.372 |  |  |  |  |
|  | Duscha et al [47] ^d^, 2018 | *P*>.05 |  |  |  |  |
|  | Frederix et al [51] ^c^, 2015 | *P*<.001 |  |  |  |  |
|  | Avila et al [60] ^c^, 2018 | *P*=.04 |  |  |  |  |
| **WC (n=5)** | | | | | | |
|  | Reid et al [41] ^d,a^, 2021 | F: *P*=.592,  M: *P*=.941 |  |  | Freene et al [67] ^d^, 2020 | *P*>.05 |
|  | Maddison et al [45] ^a^, 2019 | *P*>.05 |  |  |  |  |
|  | Prince et al [46] ^d^, 2018 | *P*=.144 |  |  |  |  |
|  | Avila et al [60] ^d^, 2018 | *P*=.11 |  |  |  |  |
| **SBP (n=10)** | | | | | | |
|  | Reid et al [41] ^d,d^, 2021 | F: *P*=.799,  M: *P*=.819 |  |  | Freene et al [67] ^b^, 2020 | *P*<.05 |
|  | Engelen et al [42] ^d^, 2020 | *P*>.05 |  |  |  |  |
|  | Claes et al [43] ^d^, 2020 | *P*=.10 |  |  |  |  |
|  | Maddison et al [45] ^a^, 2019 | *P*>.05 |  |  |  |  |
|  | Prince et al [46] ^a^, 2018 | *P*=.774 |  |  |  |  |
|  | Alsaleh et al [50] ^c^, 2016 | *P*<.05 |  |  |  |  |
|  | Frederix et al [51] ^a^, 2015 | *P*=.3 |  |  |  |  |
|  | Avila et al [60] ^a^, 2018 | *P*=.73 |  |  |  |  |
|  | Devi et al [62] ^c^, 2014 | *P*=.003 |  |  |  |  |

^a^The between-group difference or the pre-post difference is not statistically significant.

^b^The pre-post difference between or within groups is statistically significant.

^c^The difference between the intervention and control groups is statistically significant.

^d^There is a trend toward improvement, but the improvement is not statistically significant.

^e^Not available.
