# Supplementary material for: Effectiveness of eHealth Interventions on Moderate-to-Vigorous Intensity Physical Activity Among Patients in Cardiac Rehabilitation: Systematic Review and Meta-analysis
Source: J Med Internet Res. 2023 Mar 29;25:e42845. doi: 10.2196/42845 (PMC10131595; doi:10.2196/42845)
Supplement: Multimedia Appendix 7 [file jmir_v25i1e42845_app7.docx]

**Multimedia Appendix 7**

Priori determined subgroup analyses of moderate-to-vigorous intensity physical activity.

| Outcomes | Subgroup analyses | N | SMD | 95% CI | *P*^a^ | *I^2^* | Heterogeneity between subgroups |
| --- | --- | --- | --- | --- | --- | --- | --- |
| **MVPA** | | | | | | | |
|  | *Sample size* | | | | | | Q(1)=2.14, *P*=.144 |
|  | < 50 participants | 6 | 0.38 | 0.09 to 0.68 | *P*=.011 | 0% |  |
|  | ≥ 50 participants | 8 | 0.15 | 0.04 to 0.26 | *P*=.010 | 0% |  |
|  | *Measurement method* | | | | | | Q(1)=0.21, *P*=.644 |
|  | Self-reported | 6 | 0.19 | 0.02 to 0.36 | *P*=.027 | 9.4% |  |
|  | Objective | 10 | 0.14 | 0.01 to 0.27 | *P*=.040 | 0% |  |
|  | *Intervention component* | | | | | | Q(1)=0.12, *P*=.731 |
|  | Single component | 7 | 0.17 | 0.03 to 0.31 | *P*=.019 | 11.6% |  |
|  | Multi-component | 7 | 0.21 | 0.03 to 0.39 | *P*=.019 | 0% |  |
|  | *Intervention characteristic* | | | | | | Q(1)=0.54, *P*=.462 |
|  | Standardized | 8 | 0.15 | 0.03 to 0.27 | *P*=.018 | 0% |  |
|  | Tailored | 6 | 0.24 | 0.04 to 0.43 | *P*=.016 | 1.0% |  |
|  | *Intervention duration* | | | | | | Q(1)=0.32, *P*=.572 |
|  | ＜12 weeks | 3 | 0.28 | -0.10 to 0.67 | *P*=.144 | 29.1% |  |
|  | ≥ 12 weeks | 11 | 0.17 | 0.06 to 0.28 | *P*=.003 | 0% |  |
|  | *Interaction with health care professionals* | | | | | | Q(1)=0.16, *P*=.691 |
|  | No interaction | 5 | 0.14 | -0.06 to 0.34 | *P*=.173 | 0% |  |
|  | Interaction | 9 | 0.19 | 0.07 to 0.31 | *P*=.002 | 0% |  |
|  | *Control characteristic* | | | | | | Q(1)=0.38, *P*=.539 |
|  | Supervised | 2 | 0.31 | -0.13 to 0.76 | *P*=.169 | 0% |  |
|  | Unsupervised | 12 | 0.17 | 0.06 to 0.28 | *P*=.002 | 0% |  |

^a^Significance tests in which for each subgroup the null hypothesis is that SMD = 0.
