# Supplementary material for: Effectiveness of eHealth Interventions on Moderate-to-Vigorous Intensity Physical Activity Among Patients in Cardiac Rehabilitation: Systematic Review and Meta-analysis
Source: J Med Internet Res. 2023 Mar 29;25:e42845. doi: 10.2196/42845 (PMC10131595; doi:10.2196/42845)
Supplement: Multimedia Appendix 8 [file jmir_v25i1e42845_app8.docx]

**Multimedia Appendix 8**

Post hoc determined subgroup analyses without overall statistic or heterogeneity between groups of moderate-to-vigorous intensity physical activity.

| Outcomes | Intervention delivery methods | N | SMD | 95% CI | *P* | Heterogeneity |
| --- | --- | --- | --- | --- | --- | --- |
| **MVPA** |  | | | | | |
|  | Wearable devices-based | 7 | 0.27 | 0.04 to 0.50 | *P*=.022 | Q(6)=5.41, *P*=.492; *I^2^*=0% |
|  | Web-based | 7 | 0.18 | 0.03 to 0.34 | *P*=.020 | Q(6)=3.31, *P*=.769; *I^2^*=0% |
|  | Communication-based | 8 | 0.19 | 0.06 to 0.31 | *P*=.003 | Q(6)=5.93, *P*=.548; *I^2^*=0% |
