# Supplementary material for: Effectiveness of eHealth Interventions on Moderate-to-Vigorous Intensity Physical Activity Among Patients in Cardiac Rehabilitation: Systematic Review and Meta-analysis
Source: J Med Internet Res. 2023 Mar 29;25:e42845. doi: 10.2196/42845 (PMC10131595; doi:10.2196/42845)

**Multimedia Appendix 9**

Post hoc subgroup analyses without overall statistic of intervention delivery methods for the time spent on moderate-to-vigorous intensity physical activity among patients in cardiac rehabilitation.


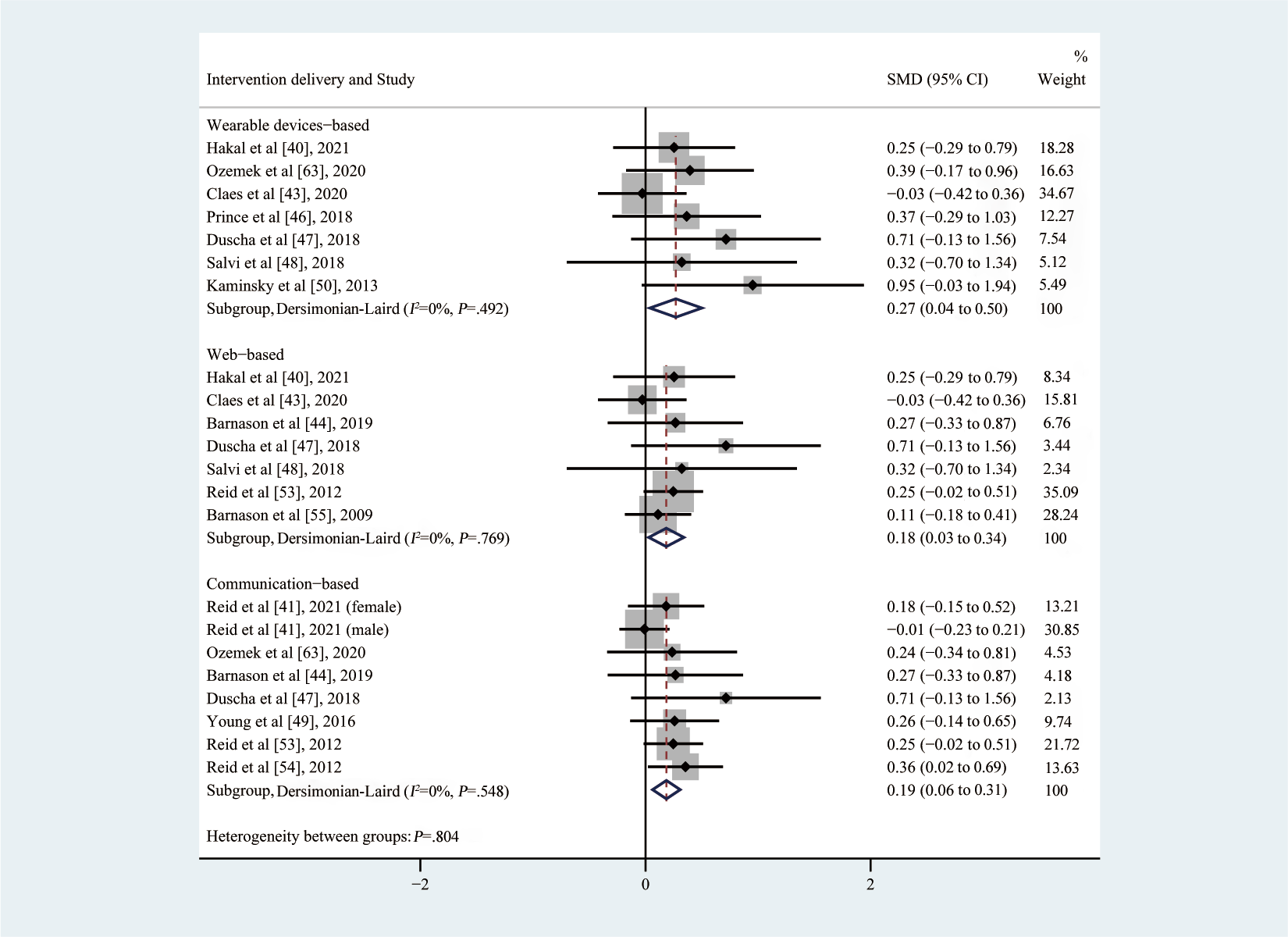

Supplement: Multimedia Appendix 9 [file jmir_v25i1e42845_app9.docx]
