# Supplementary material for: Effectiveness of eHealth Interventions on Moderate-to-Vigorous Intensity Physical Activity Among Patients in Cardiac Rehabilitation: Systematic Review and Meta-analysis
Source: J Med Internet Res. 2023 Mar 29;25:e42845. doi: 10.2196/42845 (PMC10131595; doi:10.2196/42845)
Supplement: Multimedia Appendix 10 [file jmir_v25i1e42845_app10.docx]

**Multimedia Appendix 10**

Funnel plot of effects of eHealth for changes in the time spent on moderate-to-vigorous intensity physical activity among patients in cardiac rehabilitation.

**
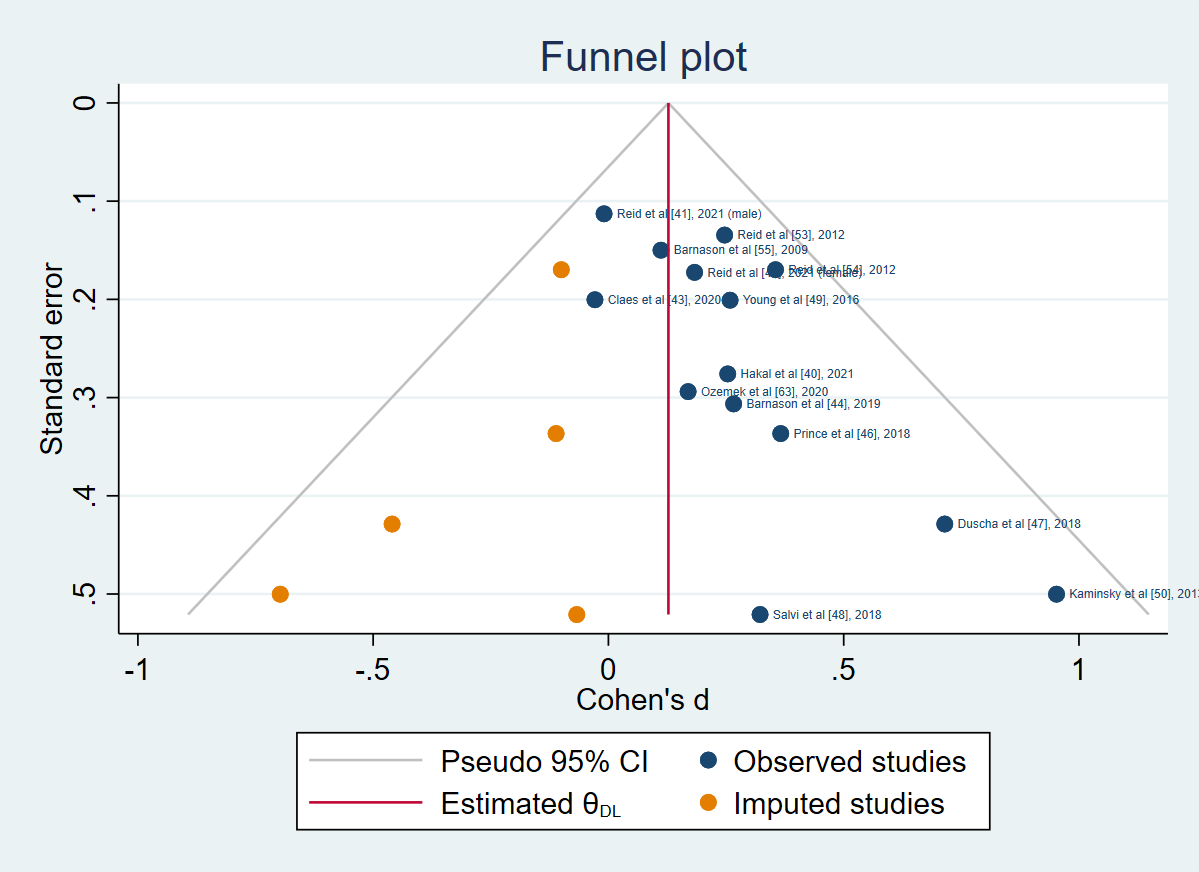
**
