# Supplementary figures and images for: Effectiveness of eHealth Interventions on Moderate-to-Vigorous Intensity Physical Activity Among Patients in Cardiac Rehabilitation: Systematic Review and Meta-analysis
Source: J Med Internet Res. 2023 Mar 29;25:e42845. doi: 10.2196/42845 (PMC10131595; doi:10.2196/42845)

**Multimedia Appendix 11**

Galbraith plot of the moderate-intensity physical activity outcome.
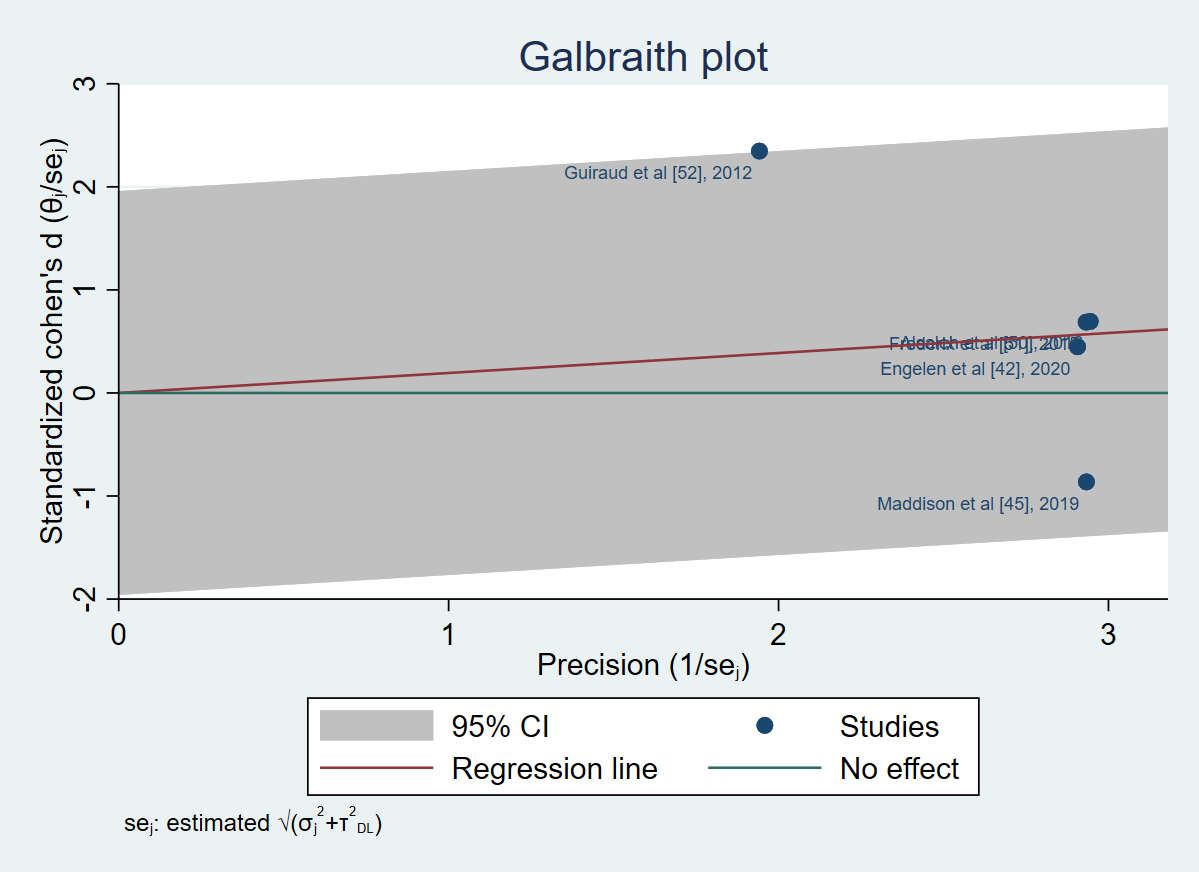

Supplement: Multimedia Appendix 11 [file jmir_v25i1e42845_app11.docx]
