# Supplementary material for: Effectiveness of eHealth Interventions on Moderate-to-Vigorous Intensity Physical Activity Among Patients in Cardiac Rehabilitation: Systematic Review and Meta-analysis
Source: J Med Internet Res. 2023 Mar 29;25:e42845. doi: 10.2196/42845 (PMC10131595; doi:10.2196/42845)
Supplement: Multimedia Appendix 12 [file jmir_v25i1e42845_app12.docx]

**Multimedia Appendix 12**

Sensitivity analyses of primary and secondary outcomes.

| Sensitivity analyses | N | SMD | 95%CI | *P* | Heterogeneity |
| --- | --- | --- | --- | --- | --- |
| **MVPA** | 14 | 0.18 | 0.07 to 0.28 | *P*=.001 | Q(13)=10.00, *P*=.694; *I^2^*=0% |
| Fixed-effects | 14 | 0.18 | 0.07 to 0.28 | *P*=.001 | Q(13)=10.00, *P*=.694; *I^2^*=0% |
| Excluding non-RCTs studies | 12 | 0.17 | 0.06 to 0.27 | *P*=.002 | Q(11)=7.59, *P*=.749; *I^2^*=0% |
| **MPA** | 5 | 0.19 | -0.12 to 0.51 | *P*=.233 | Q(4)=13.49, *P*=.009; *I^2^*=70.3% |
| Fixed-effects | 5 | 0.13 | -0.04 to 0.29 | *P*=.131 | Q(4)=13.49, *P*=.009; *I^2^*=70.3% |
| Excluding the single study with the  greater influence on the pooled  effect size | 4 | 0.30 | 0.03 to 0.56 | *P*=.030^a^ | Q(3)=5.41, *P*=.144; *I^2^*=44.6% |
| **VPA** | 3 | 0.20 | 0.00 to 0.39 | *P*=.048 | Q(2)=0.88, *P*=.643; *I^2^*=0% |
| Fixed-effects | 3 | 0.20 | 0.00 to 0.39 | *P*=.048 | Q(2)=0.88, *P*=.643; *I^2^*=0% |
| **CRF** | 7 | 0.26 | -0.04 to 0.57 | *P*=.090 | Q(6)=27.14, *P*<.001; *I^2^*=77.9% |
| Fixed-effects | 7 | 0.12 | -0.01 to 0.25 | *P*=.074 | Q(6)=27.14, *P*<.001; *I^2^*=77.9% |
| Excluding the single study with the  greater influence on the pooled  effect size | 6 | 0.35 | 0.09 to 0.62 | *P*=.009^a^ | Q(5)=11.76, *P*=.038; *I^2^*=57.5% |
| **WC** | 4 | 0.05 | -0.22 to 0.32 | *P*=.696 | Q(3)=7.13, *P*=.068; *I^2^*=57.9% |
| Fixed-effects | 4 | 0.08 | -0.08 to 0.23 | *P*=.341 | Q(3)=7.13, *P*=.068; *I^2^*=57.9% |
| **SBP** | 8 | -0.11 | -0.35 to 0.13 | *P*=.355 | Q(7)=28.40, *P*<.001; *I^2^*=75.4% |
| Fixed-effects | 8 | -0.11 | -0.23 to 0.00 | *P*=.058 | Q(7)=28.40, *P*<.001; *I^2^*=75.4% |

^a^Inconsistent with the previous result.
