# Supplementary material for: Effectiveness of eHealth Interventions on Moderate-to-Vigorous Intensity Physical Activity Among Patients in Cardiac Rehabilitation: Systematic Review and Meta-analysis
Source: J Med Internet Res. 2023 Mar 29;25:e42845. doi: 10.2196/42845 (PMC10131595; doi:10.2196/42845)
Supplement: Multimedia Appendix 13 [file jmir_v25i1e42845_app13.docx]

**Multimedia Appendix 13**

Summary of the quality of the evidence for eHealth interventions versus control.

| Certainty assessment | | | | | | | № of patients | | Effect | | Certainty | Importance |
| --- | --- | --- | --- | --- | --- | --- | --- | --- | --- | --- | --- | --- |
| № of  studies | Study design | Risk of bias | Inconsistency | Indirectness | Imprecision | Other considerations | eHealth | non-eHealth | Relative  (95% CI) | Absolute  (95% CI) |  |  |
| MVPA | | | | | | | | | | | | |
| 13 | randomized trials | not serious | not serious | not serious | not serious | publication bias strongly suspected ^a^ | 720 | 710 | - | SMD 0.18 SD higher (0.07 higher to 0.28 higher) | ⨁⨁⨁◯ Moderate | Critical |
| MPA | | | | | | | | | | | | |
| 5 | randomized trials | not serious | serious ^b^ | not serious | serious ^c^ | none | 272 | 322 | - | SMD 0.19 SD higher  (0.12 lower to 0.51 higher) | ⨁⨁◯◯  Low | Critical |
| VPA | | | | | | | | | | | | |
| 3 | randomized trials | not serious | not serious | not serious | not serious | none | 187 | 237 | - | SMD 0.2 SD higher  (0 to 0.39 higher) | ⨁⨁⨁⨁  High | Critical |
| CRF | | | | | | | | | | | | |
| 6 | randomized trials | not serious | very serious ^d^ | not serious | serious ^c^ | none | 456 | 455 | - | SMD 0.26 SD higher  (0.04 lower to 0.57 higher) | ⨁◯◯◯  Very low | Important |
| WC | | | | | | | | | | | | |
| 3 | randomized trials | not serious | serious ^b^ | not serious | serious ^c^ | none | 309 | 316 | - | SMD 0.05 SD higher  (0.22 lower to 0.32 higher) | ⨁⨁◯◯  Low | Important |
| SBP | | | | | | | | | | | | |
| 7 | randomized trials | not serious | very serious ^d^ | not serious | serious ^c^ | none | 574 | 609 | - | SMD 0.11 SD lower  (0.35 lower to 0.13 higher) | ⨁◯◯◯  Very low | Important |

^a^Downgraded one level for publication bias: the funnel plot is not symmetrical and the Eggers’ test is statistically significant (*P*=.01).

^b^Downgraded one level for heterogeneity: the *P* value obtained by the heterogeneity test is small and the square of the *I* statistic value is statistically significant.

^c^Downgraded one level for imprecision: the confidence interval contains the invalid value.

^d^Downgraded two levels for heterogeneity: the *P* value obtained by the heterogeneity test is very small and the square of the *I* statistic value is very large.
