# Supplementary material for: Effectiveness of eHealth Interventions on Moderate-to-Vigorous Intensity Physical Activity Among Patients in Cardiac Rehabilitation: Systematic Review and Meta-analysis
Source: J Med Internet Res. 2023 Mar 29;25:e42845. doi: 10.2196/42845 (PMC10131595; doi:10.2196/42845)
Supplement: Multimedia Appendix 14 [file jmir_v25i1e42845_app14.docx]

**Multimedia Appendix 14**

Definitions, measures, and findings of adherence to eHealth interventions.

| Studies | Definition of adherence | Measure of adherence | Findings |
| --- | --- | --- | --- |
| Hakal et al [40], 2021 | Subjective adherence to the treatment. | A separate questionnaire. | The results from a subjective questionnaire indicated that on a scale from 1 to 7, in which 1 indicated “totally agree” and 7 indicated “totally disagree”, the participants almost or somewhat agreed they had actively used the Fitbit Charge HR (mean 2.4, SD 2.0). |
|  | Adherence on using internet software. | The number of recordings made to the software, and the number of messages sent to the care provider. | Participants in the experimental group made an average of 98 (167) recordings regarding their PA in Movendos mCoach and sent an average of 6.4 (6) messages to the care provider. |
| Reid et al [41], 2021 | Intervention adherence. | 9 scheduled Exercise Facilitator Intervention (EFI) sessions completions. | Women completed an average of 6.4 (2.3) of 9 scheduled EFI sessions and men completed an average of 6.8 (1.9). For female and male participants, 48/68 (70.6%) and 123/158 (77.8%), respectively, completed ≥ 6 of the 9 scheduled treatment sessions. |
| Claes et al [43], 2020 | Adherence to PATHway intervention. | Weekly intervals of the combined upload frequency of Exerclasses, Exergames, and Active Lifestyle activities. | Active lifestyle recorded by means of the Microsoft band 2 (median 27, range 2.5-89.5), Exerclasses (median 14.5, range: 3-35.8), and Exergames (median 1, range: 0-3). |
| Maddison et al [45], 2019 | Exercise adherence. | The completion of prescribed exercise sessions (maximum=36). | At 12 weeks, exercise adherence (n/36) was 21 (13) in the REMOTE-CR group and 23 (11) in the center-based group, and the adjusted mean difference (95% CI) was −1.97 (−5.74 to 1.81). |
| Salvi et al [48] 2018 | Adherence during the rehabilitation program. | Comparison of the total number of prescribed exercise sessions and the number of sessions that were actually started by the patient. | Because of the Internet connectivity problems, exercise data was received from 25 patients of 40 who actually started the rehabilitation program. Of these, 17 were among those who completed the study. Of the 25 people for whom data were collected, the number of sessions actually started was 61% (CI: 15%) of the total number of prescribed exercise sessions; 79% (CI: 12%) of the 17 people who completed this study. |
|  |  | The percentage of exercise sessions explicitly cancelled by users. | The percentage of exercise sessions cancelled was 4% (CI: 3%) of the 25 people for whom data were collected; 2% (CI: 1%) of the 17 people who completed this study. |
|  |  | Comparison of the total number of minutes of exercise prescribed and the number of minutes of exercise that were actually performed by the patient. | Of the 25 people for whom data were collected, the number of minutes of exercise actually performed was 32% (CI: 12%) of the prescribed minutes of exercise; 45% (CI: 14%) of the 17 people who completed this study. |
| Young et al [49], 2016 | Adherence of exercising regularly. | 80% of the recommended 150-min per week of moderate or higher intensity activity. | 8.2% (4/49) participants of the intervention group met at baseline, 12% (6/50) met at 3 months, and 8.5% (4/47) met at 6 months. 0% (0/42) participants of the control group met at baseline, 4.3% (2/46) met at 3 months, and 4.3% (2/47) met at 6 months. |
| Alsaleh et al [50], 2016 | Adherence to completion of PA. | PA diaries. | Sixty-five (92 %) of intervention participants completed at least one goal in the PA diary, and 66 (93 %)  completed diaries for all 12 weeks of the intervention period. Adherence to completion of PA diaries dropped 3% across the intervention period. |
| Guiraud et al [52], 2012 | Adherence to PA. | The percentage of patients reaching more than 150-min, more than 120-min, more than 90-min and less than 90-min of MPA per week. | The percentage of patients reaching more than 150-min of MPA per week increased from 15.7% to 36.8% between the 1st and 8th week of follow-up. Who completed a level close to the recommendations for PA (more than 120-min), from 26.3% to 57.9%. An increase in those who performed more than 90-min of MPA per week from 47.3% to 68.5%. In contrast, there was a decrease in the number of patients engaged in MPA lasting less than 90 minutes, from 52.7% to 31.5%. |
| Reid et al [53], 2012 | Adherence to allocated intervention. | The number of online tutorials completed. | The mean number of online tutorials completed by CardioFit participants was 2.7 of a maximum five, and 61.7% of participants completed at least three of the five tutorials. Completion rates for the tutorials at 2, 4, 8, 14, and 20 weeks were 70, 64, 56, 47, and 43%, respectively. |
|  |  | The number of emails sent to exercise specialist. | Thirty-seven CardioFit participants emailed the exercise specialist at least once. Of those emailing the specialist at least once, the average number of emails was 3.3 (range 1-8). |
| Reid et al [54], 2012 | Adherence to motivational counselling intervention. | Completion rates for scheduled sessions | Completion rates for scheduled sessions at time 0 and 2, 4, 8, 14, 20, 24, 40, and 52 weeks were 100, 94, 91, 91,90, 87, 88, 86, and 83%, respectively. |
| Pinto et al [65], 2022 | Adherence to the program. | The number of online exercise training sessions. | Nearly half (46.9%) of the participants did at least one online exercise training session per week. Among those who did training sessions, 58% did two or three times per week, 27% once per week and 15% more than four times per week. |
|  |  | The number of online educational sessions. | 49% of the participants attended at least one of the 13 online educational sessions. |
| Freene et al [67], 2020 | Adherence to the program. | The completion of Do’s as marked by the participant. | 73.7% (252/342) of the Do’s sent to participants during the 6-week intervention period were marked as completed. |
| Antypas et al [59], 2014 | Adherence to the website | Duration of use of website in days. | At 1 year from baseline, the adherence rate was 25.6% for the tailored group and 24.0% for the controls. The median for adherence time for the tailored group was 45.0 (95% CI: 0.0-169.8) days and 111.0 (95% CI:  45.1-176.9) days for the control group; these findings were not significantly different (*P* = 0.39). The median adherence time for men was 122.0 (95% CI: 14.8-229.2) days and 75.0 (95% CI: 0.0-153.3) days for women; these values were significantly different (*P* = 0.04). |
| Devi et al [62], 2014 | Adherence to the Rehabilitation Program. | Completion of the 4 stages of improving patients’ cardiac risk profile within 6 weeks. | Of the 48 intervention group participants, 19 (40%) completed the intervention and 29 (60%) did not progress past stage 3. |
